# Charcot-Marie-Tooth–Linked Mutant GARS Is Toxic to Peripheral Neurons Independent of Wild-Type GARS Levels

**DOI:** 10.1371/journal.pgen.1002399

**Published:** 2011-12-01

**Authors:** William W. Motley, Kevin L. Seburn, Mir Hussain Nawaz, Kathy E. Miers, Jun Cheng, Anthony Antonellis, Eric D. Green, Kevin Talbot, Xiang-Lei Yang, Kenneth H. Fischbeck, Robert W. Burgess

**Affiliations:** 1 The Jackson Laboratory, Bar Harbor, Maine, United States of America; 2 Neurogenetics Branch, National Institute of Neurological Disorders and Stroke, National Institutes of Health, Bethesda, Maryland, United States of America; 3 MRC Functional Genomics Unit, University of Oxford, Oxford, United Kingdom; 4 The Scripps Research Institute, La Jolla, California, United States of America; 5 Genome Technology Branch, National Human Genome Research Institute, National Institutes of Health, Bethesda, Maryland, United States of America; 6 Department of Human Genetics, University of Michigan Medical School, Ann Arbor, Michigan, United States of America; 7 Department of Neurology, University of Michigan Medical School, Ann Arbor, Michigan, United States of America; 8 Department of Clinical Neurology, University of Oxford, John Radcliffe Hospital, Oxford, United Kingdom; Stanford University School of Medicine, United States of America

## Abstract

Charcot-Marie-Tooth disease type 2D (CMT2D) is a dominantly inherited peripheral neuropathy caused by missense mutations in the glycyl-tRNA synthetase gene (*GARS*). In addition to *GARS*, mutations in three other tRNA synthetase genes cause similar neuropathies, although the underlying mechanisms are not fully understood. To address this, we generated transgenic mice that ubiquitously over-express wild-type *GARS* and crossed them to two dominant mouse models of CMT2D to distinguish loss-of-function and gain-of-function mechanisms. Over-expression of wild-type *GARS* does not improve the neuropathy phenotype in heterozygous *Gars* mutant mice, as determined by histological, functional, and behavioral tests. Transgenic *GARS* is able to rescue a pathological point mutation as a homozygote or in complementation tests with a *Gars* null allele, demonstrating the functionality of the transgene and revealing a recessive loss-of-function component of the point mutation. Missense mutations as transgene-rescued homozygotes or compound heterozygotes have a more severe neuropathy than heterozygotes, indicating that increased dosage of the disease-causing alleles results in a more severe neurological phenotype, even in the presence of a wild-type transgene. We conclude that, although missense mutations of *Gars* may cause some loss of function, the dominant neuropathy phenotype observed in mice is caused by a dose-dependent gain of function that is not mitigated by over-expression of functional wild-type protein.

## Author Summary

Mutations in the glycyl-tRNA synthetase gene (*GARS)* cause Charcot-Marie-Tooth disease type 2D, a disease characterized by neuronal axon loss in the arms and legs, resulting in weakness and sensory problems. The GARS protein is essential for protein synthesis in every cell, and it has been difficult to determine whether the mutations result in disease because they impair this function or whether GARS somehow becomes toxic when it is mutated. We generated mice that overexpress normal GARS and mated these to two different mouse models of the disease to determine whether a restoration of normal function could prevent the disease. These crosses demonstrated that the mutant forms of GARS are toxic, and this toxic effect increases as the amount of mutant protein increases. Furthermore, this toxicity cannot be reduced or prevented by providing additional normal GARS. Therefore, these results suggest that, for most patients, therapies need to specifically target the mutant form of *GARS* or the toxic function.

## Introduction

Charcot-Marie-Tooth disease (CMT) is a heterogeneous group of inherited neuropathies affecting approximately 1 in 2,500 people [1]. CMT is divided into type 1 forms, characterized by demyelination and decreased nerve conduction velocity, type 2 or axonal forms, characterized by axon loss and reduced evoked potential amplitudes, and intermediate forms having features of both demyelinating and axonal neuropathy [2], [3].

As the genetic causes of CMT have been identified, shared mechanisms have been elucidated that help explain the myelin degeneration in CMT type 1 and axon degeneration in CMT type 2. Disruption of myelin structure through mutation of proteins produced by peripheral Schwann cells is a recurring mechanism in type 1 CMTs [4]–[6]. Defects associated with vesicle trafficking [7], [8], mitochondrial morphology [9]–[11], and cytoskeletal integrity [12], [13] have each been implicated in multiple axonal forms of CMT.

Other axonal and intermediate CMTs have been linked to mutations in tRNA synthetase genes. The first to be identified and best characterized is CMT type 2D, caused by mutations in the glycyl-tRNA synthetase gene (*GARS*, MIM ID 601472) [14]. Subsequently, mutations in tyrosyl-tRNA synthetase (*YARS*, MIM ID 608323) were identified in patients with dominant intermediate CMTC [15]; a single missense change in the alanyl-tRNA synthetase gene (*AARS*, MIM ID 613287) was associated with axonal CMT2N [16]; and most recently compound heterozygous frame shift/missense mutations in the lysyl-tRNA synthetase gene (*KARS*, MIM ID 613641) were reported in a patient with recessive intermediate CMTB [17]. While human genetic studies have implicated mutations in multiple tRNA synthetase genes, the pathogenic mechanism for these neuropathies is still unclear, although a shared mechanism is an attractive hypothesis [18]. In particular, it is unclear whether the mutations result in a pathological gain of function, a partial loss of activity related to translation, an impact on an unknown, noncanonical activity, or a combination of these mechanisms.

tRNA synthetases covalently link tRNAs with their cognate amino acids to translate the genetic code. These enzymes serve an essential, nonredundant role in protein synthesis. It has been suggested that a reduction of this tRNA-charging function could result in neuropathy, and peripheral nerves with long, large-diameter axons may be especially vulnerable to decreased activity because of their unusual transport and metabolic demands [15], [19]. Consistent with this, several disease-causing mutations in *GARS* are associated with a reduction in charging function in cell-free assays, and this is roughly correlated with a decreased ability to rescue nonviability in yeast caused by deficiency of *GRS1*, the ortholog of *GARS*. However other mutations do not impair this function [19]–[21].

Structural studies have shown that many of the mutations alter dimer association, affecting homodimer formation that is essential for tRNA-charging activity [20], [22]. Furthermore, all mutations tested alter the subcellular distribution of GARS in transfected cells, also suggesting a possible loss of function at the cellular level through mislocalization, even if charging activity is preserved [19]–[21], [23], [24].

Two mouse models of CMT2D that share pathological features with the human disease, with differing severity, are caused by dominant amino acid substitutions in *Gars*. The *Gars^Nmf249^* allele (hereafter abbreviated *Nmf249*) causes reduced body weight and impaired mobility in heterozygous mice [25]. Axon number and neuromuscular junction (NMJ) morphology are normal at post-natal day 7, but subsequently axons are lost without a reduction in myelin thickness; NMJs show partial and sometimes complete denervation. The *Nmf249* allele is an insertion in the *Gars* gene that substitutes lysine and tyrosine for proline at position 278 in the mouse GARS protein, equivalent to a P234KY change in human GARS (Note: numbering differences are because the human annotation does not consider the N-terminal mitochondrial localization signal appended through alternative start codon usage).

The *Gars^C201R^* allele (hereafter abbreviated *C201R*) is less severe and was identified in a chemical mutagenesis screen. In addition to impaired grip strength, these mice have impaired motor control, diminished muscle force, reduced weight, a shift towards smaller axon diameters, and some muscle denervation [26]. The mouse C201R substitution is equivalent to C157R in human GARS. These two alleles demonstrate the spectrum of phenotypic severity in mice and provide a range of tests that can be used to quantify the effects on neuromuscular function.

Previous studies in mice are consistent with either a pathological gain-of-function for the mutant protein, or a partial loss-of-function, or both. Mouse and human mutations both cause dominantly inherited neuropathy. Moreover, mice heterozygous for a gene trap allele (*Gars^XM256^* ^/+^, a presumed null allele, hereafter abbreviated *XM256*) do not have a phenotype, arguing against a simple loss-of-function and ruling out haploinsufficiency as a cause of the neuropathy phenotype [25]. As expected for a gene with such a critical activity, homozygous gene trap mice die as embryos. Mice homozygous for the *C201R* allele are more severely affected than heterozygotes of either mutant allele and die at about two weeks of age [26]. This increased severity may be the result of doubling a pathological gain of function associated with the C201R protein, or the inability of the mutant protein to actively perform its normal function, or a combination of these effects. Consistent with the inability of the mutant protein to sustain its normal function, both the *C201R* and the *Nmf249* mutations fail to complement the loss-of-function *XM256* allele, resulting in embryonic death in the absence of wild-type protein, without an increase in the genetic dosage of the mutant allele [25], [26]. The embryonic death of these mice may indicate that wild-type GARS is mitigating a toxic gain of function, or that the mutant proteins do not retain their normal function, or it may indicate a combination of these mechanisms.

We hypothesized that a partial loss-of-function could be corrected by transgenic over-expression of a functional wild-type GARS protein, whereas a pathological gain-of-function could be independent of wild-type activity and therefore would not necessarily be corrected by increased wild-type expression. In order to determine whether increased wild-type GARS rescues the *Gars* mutant mice, we crossed transgenic mice that over-express the wild-type human *GARS* gene to both the *Nmf249/+* and *C201R/+* mice, and analyzed the effects on the phenotype. We found a recessive reduction in perinatal viability in *C201R* as a homozygote or in combination with the *XM256* gene trap allele, which could be rescued by wild-type *GARS* over-expression. However, wild-type over-expression did not substantially mitigate the neuropathy phenotype in heterozygous *Nmf249/+* or *C201R/+* mice, consistent with a predominant gain of function as the cause of peripheral neuropathy in these mice.

## Results

### Transgenic mice over-expressing human *GARS*

**Figure 1:**
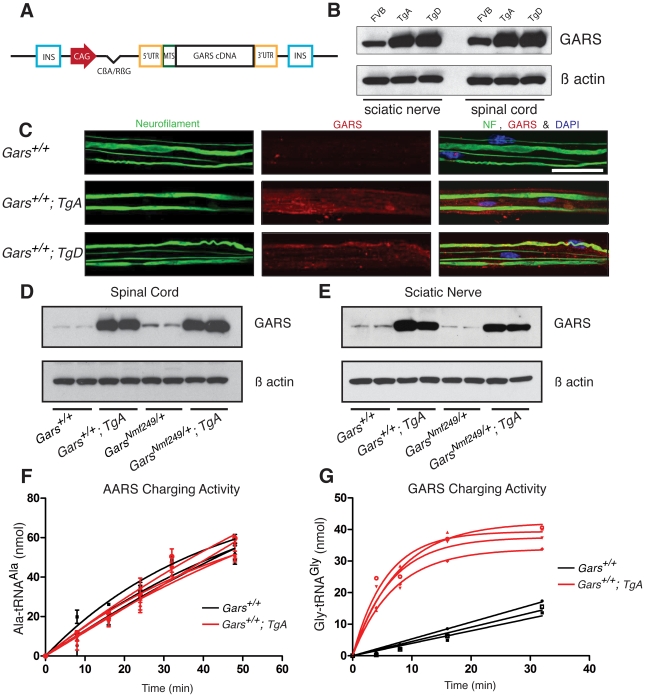
The *CAG-GARS* transgenic construct is robustly expressed in tissues and cell types affected by CMT. (A) The wild-type *GARS* transgene construct contains a *CAG* promoter, followed by an intron, and the full-length *GARS* cDNA, including the N-terminal mitochondrial localization signal, with flanking 5′ and 3′ UTR sequence. These elements were between two insulator sequences (INS). (B) Immunoblotting with anti-GARS confirmed that transgenes A and D express at similar levels, both above the level of FVB/N wild-type mice in spinal cord and sciatic nerve. Anti-β actin was used as a loading control. (C) Confocal images of teased fiber preparations from sciatic nerve stained with neurofilament and GARS antibodies from one-month-old animals. All three red channel images (GARS staining) were matched for laser intensity, gain, and the Z depth of field (scale bars are 40 µm). To confirm that transgenes A and D were overexpressed in these tissues, exposure-matched analysis was performed on FVB/N control mice (n = 3), *TgA* mice (n = 3) and *TgD* mice (n = 3). F1 mice with the transgenes overexpress GARS in sciatic nerve and spinal cord. We crossed *Nmf249/+* mice with WT; *TgD* mice and analyzed F1 progeny in all of the four resulting genotypes. Western blot was used to evaluate expression in the spinal cord (D) and in the sciatic nerve (E). On both wild-type and mutant backgrounds, the transgene overexpresses wild-type GARS in these tissues. F) Aminoacylation activity for alanyl tRNA synthetase (AARS) was not different between wild type (black) and WT;TgA (red) spinal cord homogenates. G) Spinal cord homogenates from WT;TgA mice showed much higher (>10 fold) aminoacylation activity when assayed for GARS activity. Three wild type and four TgA littermates were tested at nine weeks of age.

Wild-type human *GARS* was expressed in mice using a transgene construct with the full-length human *GARS* open reading frame under the control of a CAG promoter [27], which is a fusion of the CMV early enhancer and the chicken β actin promoter and leads to strong ubiquitous transcription of downstream elements (Figure 1A). Two independent transgene founder strains, designated Transgene A (*TgA*) and Transgene D (*TgD*), were identified and used in this study. These two lines over-express GARS in spinal cord and sciatic nerve at similar levels (>10 times endogenous levels), as measured by immunoblotting (Figure 1B).

### Transgenic *GARS* is expressed in myelinating Schwann cells and sciatic nerve axons

In order to examine *in situ* expression of the transgenic protein in relevant cell types, we excised and fixed the sciatic nerve and teased the fibers apart to improve antibody penetration and to allow visualization of individual axons.

Endogenous GARS is present at low levels in the sciatic nerve (Figure 1C and [21]). In exposure-matched confocal images, anti-GARS staining was increased in animals with either *TgA* or *TgD* over the endogenous levels in strain matched FVB/N control mice without the transgene. The pattern of GARS immunoreactivity in transgenic mice was also similar to that of wild type mice, with staining found in both the axon and Schwann cells, suggesting the transgenic protein localizes normally (Figure 1C). Therefore, the transgenes produce protein that is present in Schwann cells and transported to peripheral axons, as indicated by overlapping neurofilament staining.

### Over-expression of wild-type *GARS* does not reduce axon loss in *Nmf249/+* mice

To examine the effect of over-expressing wild-type *GARS* on the neuropathy phenotype, the more severe *Nmf249/+* mice were crossed with hemizygous transgenic mice and the four resulting F1 genotypes were analyzed: wild type, WT; *Tg*, *Nmf249/+*, and *Nmf249/+; Tg*. The mice were analyzed at post-natal day 30 (P30). Mice with the transgene had much stronger levels of GARS protein expression than non-transgenic littermates in spinal cord and sciatic nerve on immunoblots of animals with both wild-type and *Nmf249/+* backgrounds (Figure 1D, 1E). Transgene expression was confirmed by immunoblot in mice with each genotype for crosses with both *Nmf249/+* and *C201R/+* (Figure S1). There was no difference overall in GARS levels between wild type and *Nmf249/+* or *C201R/+*, however, both transgenic strains result in expression well above endogenous levels. Consistent with the increased GARS protein, aminoacylation activity was also increased, indicating the transgenic protein is enzymatically active. Homogenates from the spinal cord of wild type and WT;TgA and TgD mice were prepared and assayed for the activity of both GARS and alanyl tRNA synthetase (AARS) as an internal control. As anticipated, no increase in activity was seen for AARS (Figure 1F), whereas GARS activity was increased at least 10 fold (as judged by the initial rate of tRNA glycylation) in each transgenic strain (Figure 1G and data not shown).

**Figure 2:**
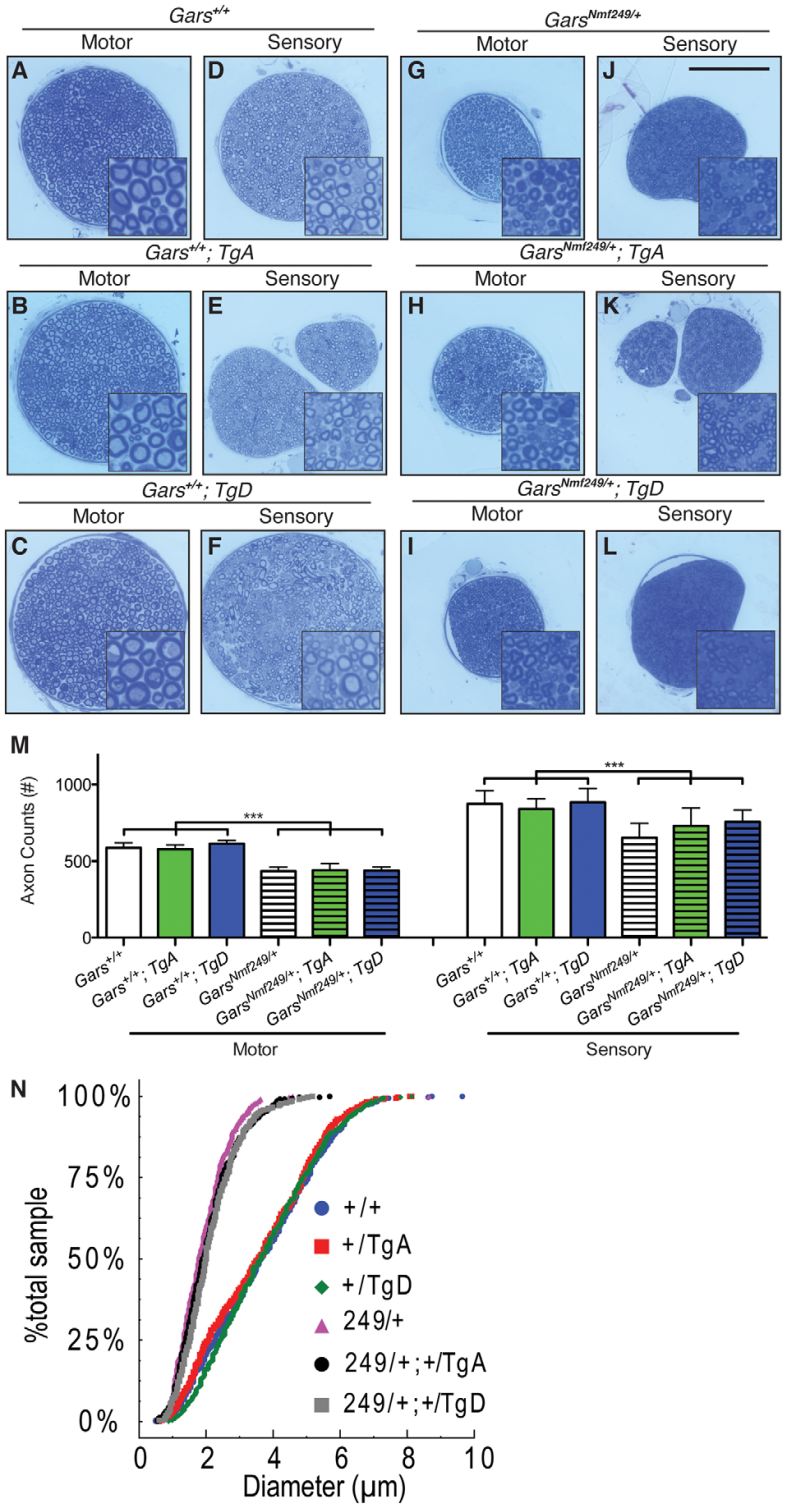
Neither motor nor sensory axon loss in *Nmf249/+* mice is abated by transgenes A or D. Semi-thin sections of motor and sensory branches of the femoral nerve collected from one month old mice were stained with Toluidine Blue. Representative images are shown in (A-L)(scale bar in J is 100 µm and higher magnification insets are 30×30 µm). (M) Myelinated axons were counted in femoral nerves. Axon counts in the motor nerves were decreased from 587±32 (n = 12) in wild type mice to 435±26 (n = 9) in *Nmf249/+* (p<0.001). Wild type mice with *TgA* and *TgD* had motor axon counts of 579±29 (n = 11) and 614±22, respectively and were not statistically different from counts in wild type mice. *Nmf249/+* mice with *TgA* had motor axon counts of 441±43 (n = 5) and with *TgD* had motor axon counts of 438±52 (n = 5), which were not significantly different compared to *Nmf249/+* mice. Mice with the mutant allele all had significantly reduced motor axon counts when compared to any of the wild type groups (p<0.001). Myelinated axon numbers in the sensory nerves were significantly (p<0.001) decreased from 876±83 (n = 12) for wild type mice to 654±94 (n = 9) for *Nmf249/+*. Wild type mice with *TgA* and *TgD* had sensory axon counts of 842±64 (n = 11) and 885±89 (n = 6), respectively, and were not statistically different from wild type mice. *Nmf249/+* mice with *TgA* had motor axon counts of 730±118 (n = 5), *Nmf249/+* mice with *TgD* had motor axon counts of 758±77 (n = 5). Transgenes A and D did not significantly alter sensory axon number compared to *Nmf249/+* alone, but were somewhat intermediate with *Nmf249/+; TgA* mice being modestly reduced relative to wild type controls (p<0.05), whereas *TgD* mice were not different from controls. (N) A cumulative histogram shows the distribution of axon diameters in all six genotypes and demonstrates that there is a shift to smaller axons in the mutant genotypes. There is an obvious clustering of WT, WT; *TgA*, and WT; *TgD* mice and *Nmf249/+, Nmf249/+*; *TgA*, and *Nmf249/+*; *TgD* mice.

To assess the effect of the transgenic *GARS* on peripheral neuropathy, we examined the femoral nerve, which consists of a primarily motor branch innervating the quadriceps, and a primarily sensory branch that becomes the saphenous nerve and innervates the skin of the lower leg. In *Nmf249/+* mice, both branches had a reduction in myelinated axon number, whereas *C201R/+* mice had normal axon numbers [25], [26]. We isolated motor and sensory branches of the femoral nerve from F1 progeny from our cross between *Nmf249/+* mice and *GARS* wild-type transgenic mice at one month of age. The nerves were fixed and embedded, and semi-thin sections were stained with toluidine blue for quantification of axon number and size (Figure 2A–2L). In these cross sections, the size of the femoral nerves was reduced in the presence of the *Nmf249/+* allele (Figure 2A–2F compared to Figure 2G–2L). This was quantified by counting myelinated axons, which showed on average a 26% reduction in axons in motor nerves with the *Nmf249/+* allele compared to wild-type littermates. There was no significant improvement in the *Nmf249/+* axon numbers in motor or sensory nerves in mice with *TgA* or *TgD* (Figure 2M).

We next examined the distribution of motor axon diameters to determine whether there was any improvement in motor nerve pathology that was not detected with the axon counts. A cumulative histogram of the frequency of observed axon diameters shows the distribution and range of axon diameters in this study. The plot shows a marked shift from large diameter axons (4–6 µm) to small diameter axons (0–3 µm) in *Nmf249/+* mice (Figure 2N). Both *TgA* and *TgD* showed small but significant improvements in axon diameter in *Nmf249/+* mice by a nested-ANOVA analysis (p = 0.006). There was no impact of either transgene on axon diameter in an otherwise wild-type background. With and without the transgene, the *Nmf249* allele significantly reduced mean axon diameter, while the proportional observed decrease in myelin thickness is not as dramatic (Figure S2).

### Nerve conduction and body weight are not improved by over-expression of wild-type *GARS*

**Figure 3:**
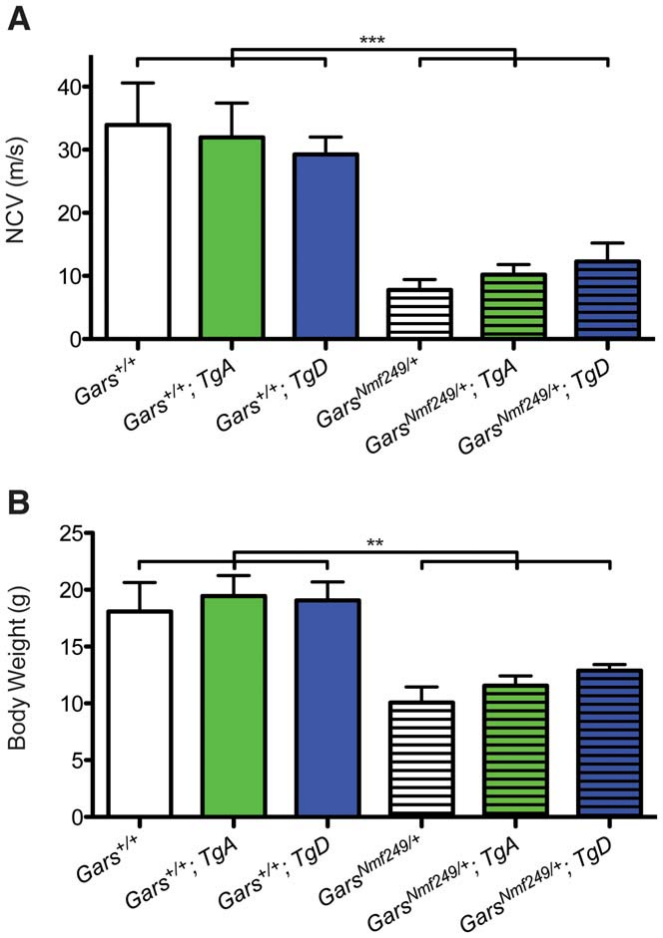
Wild-type over-expression does not improve motor nerve conduction and body weight in *Nmf249/+* mice. (A) The *Nmf249/+* allele significantly reduces nerve conduction velocity (NCV) in one-month-old mice from 33.9±6.7 m/s (n = 8) measured in control wild-type animals to 7.8±1.7 m/s (n = 7) (p<0.001). *Nmf249/+*; *TgA* had nerve conduction of 10.2±1.6 m/s (n = 5) and *Nmf249/+*; *TgD* had velocities of 12.3±3.0 m/s (n = 3), which were not significantly different than *Nmf249/+*. Compared to wild-type, all three mutant genotypes had significantly reduced NCVs (p<0.001). WT; *TgA* and WT; *TgD* NCVs were not significantly different from wild-type mice: 32.0±5.4 (n = 8) and 29.3±2.8 (n = 6), respectively. (B) As an indicator of overall health, body weight was measured at P30. Wild-type mice weighed 18.1±2.6 g (n = 8) and were not significantly different from WT; *TgA* mice, which weighed 19.5±1.8 g (n = 6), and WT; *TgD* mice that weighed 19.0±1.6 g (n = 6). Compared to wild type mice, *Nmf249/+* mice were significantly lighter, weighing 10.1±1.4 g (n = 8)(p<0.001). *Nmf249/+*; *TgA* and *Nmf249/+*; *TgD* mice were not significantly different than nontransgenic mutant mice with a mean weight of 11.6±0.8 g (n = 5) and 12.9±0.5 g (n = 3) respectively.

Consistent with the slight improvement in axon diameters observed in *Nmf249/+* mice with *TgA* and *TgD* (Figure 2N), there was trend towards improvement in nerve conduction velocity, but this was not significant. Nerve conduction in *Nmf249/+* mice is significantly reduced to approximately 25% of wild-type velocities. *Nmf249/+*; *TgA* and *Nmf249/+*; *TgD* mice had NCVs that did not differ significantly from *Nmf249/+* alone (Figure 3A).

Previous studies show that between 2 and 4 weeks of age, mice with the *Nmf249/+* allele stop gaining weight and become significantly smaller than littermate controls [25]. Various factors are likely to contribute to the decreased weight. Denervation decreases muscle mass and probably compromises ability to feed. As a measure of overall health, we weighed the mice in this study. Consistent with measures of peripheral nerve function, mutant mice with *TgD* trended toward being heavier, but neither *TgA* nor *TgD* significantly improved the body weights of *Nmf249/+* mice, and all mutant animals were still significantly lighter than littermate control mice (Figure 3B).

### *GARS* over-expression does not improve neuromuscular junction occupancy in CMT2D mice

The loss of motor axons in peripheral nerves of *Nmf249/+* mice also results in a disruption of the neuromuscular junction (NMJ). Mature, healthy motor neurons innervate muscle with pretzel-shaped presynaptic terminals. During development, post-synaptic regions mirror the shape of the axon terminal in regions of specialization where acetylcholine receptors concentrate to define the muscle side of the NMJ. The loss of distal motor axons is reflected in the NMJs of *Nmf249/+* mice, which are severely dysmorphic. Many junctions in mutant mice are partially or completely denervated by post-natal day 30 [25]. To further study whether over-expression of *GARS* affects the motor phenotype in CMT2D mice, we looked at NMJ morphology and occupancy.

**Figure 4:**
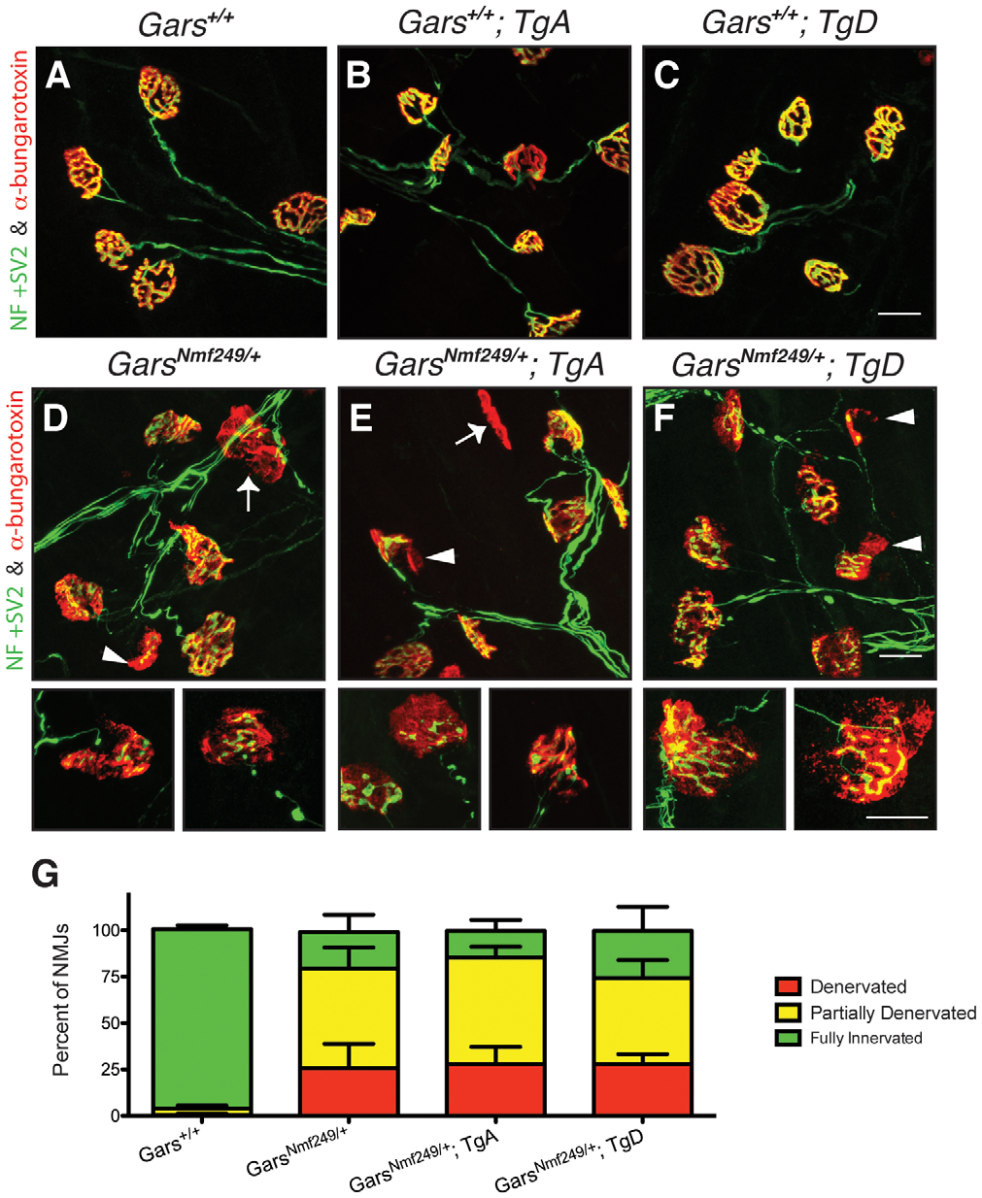
Neuromuscular junction morphology and occupancy. Neuromuscular junctions (NMJs) from plantaris muscles were collected from one-month-old mice and visualized with antibodies labeling neurofilament (NF) and synaptic vesicle protein 2 (SV2) (green), and α-bungarotoxin, which binds acetylcholine receptors (AChRs) (red). NMJs from (A) wild type, (B) WT; *TgA*, and (C) WT; *TgD* mice were fully occupied by presynaptic terminals that completely overlap signals from postsynaptic markers with a complex, pretzel-like morphology. D) *Nmf249/+*, (E) *Nmf249/+*; *TgA*, and (F) *Nmf249/+*; *TgD* mice have NMJs with regions of post-synaptic staining that are not innervated (arrowheads) and some post-synaptic NMJs have no associated nerve (arrows). (G) The occupancy of NMJs was quantified by scoring as fully innervated, partially denervated or fully denervated, based on the overlap between pre- and post-synaptic staining. 96.7±2.1% of wild-type NMJs were fully innervated, while only 19.6±9.3% were fully innervated in *Nmf249/+* mice (p<0.001). No significant change was seen between mutant mice and mutant mice with transgenes A and D, where 14.5±5.9% and 25.5±12.8% of NMJs were fully innervated, respectively. All scale bars are 20 µm.

NMJs from wild-type mice with and without the transgene had normal “pretzel-like” morphology with full overlap between pre- and post-synaptic terminals (Figure 4A–4C). In mutant mice (Figure 4D), there are regions where the presynaptic markers are absent in areas where there is post-synaptic staining (arrowheads). There are also post-synaptic junctions that have no associated nerve terminal, indicating that the muscle fiber has been denervated (arrows). The morphology and occupancy of NMJs in *Nmf249/+*; *TgA* mice (Figure 4E) and *Nmf249/+*; *TgD* (Figure 4F) were not improved. In each animal, NMJs were scored based on the overlap between pre- and post-synaptic markers and classified as fully innervated, partially denervated, or fully denervated. This assessment of NMJ occupancy showed there was no significant improvement in mutant mice with transgenes over mutant mice without transgenes (Figure 4G). Therefore, with the exception of a slight increase in axon diameters, the over-expression of wild type *GARS* as a transgene did not lead to significant improvements in any phenotypic measure in the *Nmf249/+* mice.

### Neither wild-type transgene improves behavioral deficits in *C201R/+* mice

**Figure 5:**
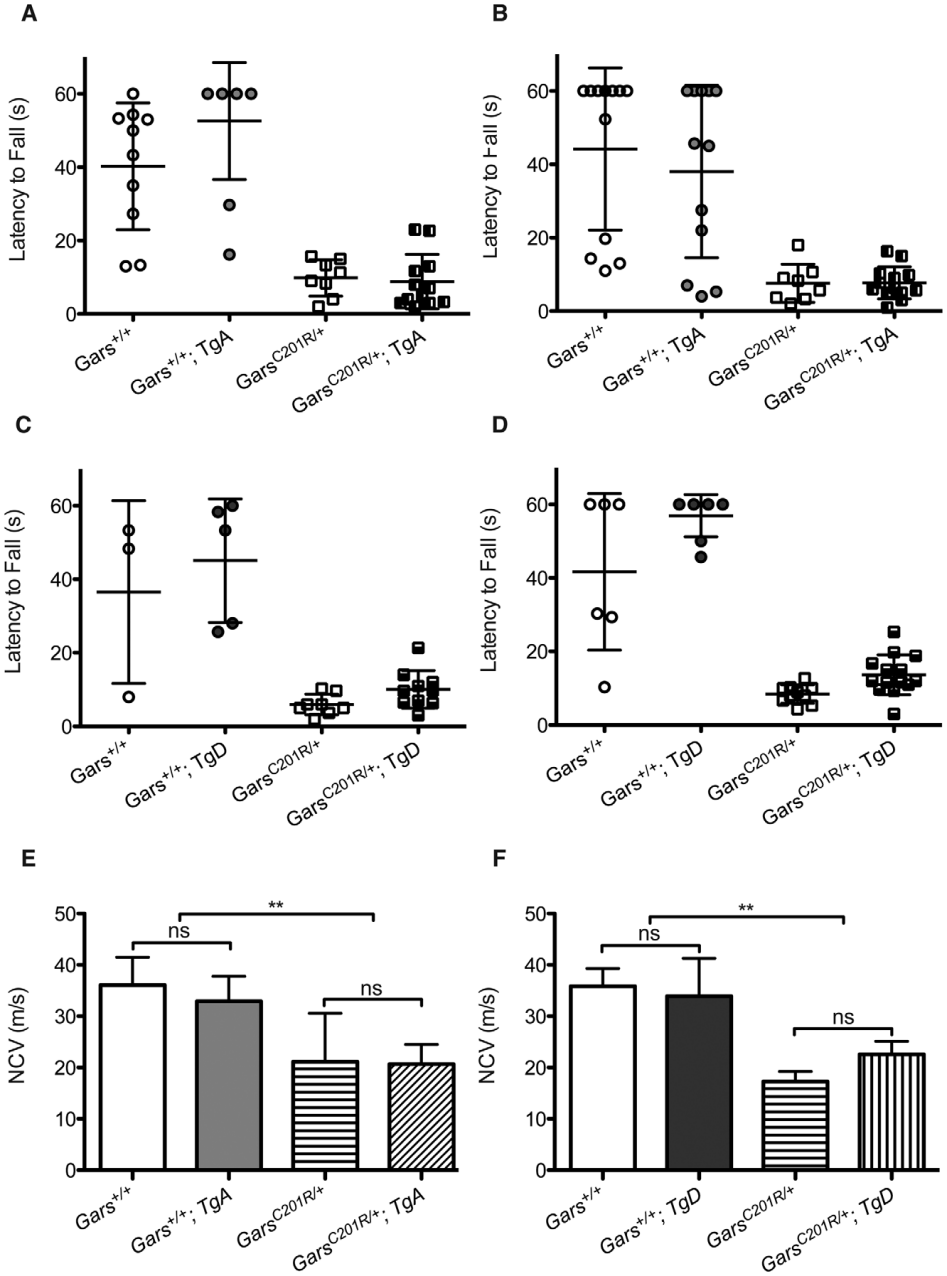
Wire hanging deficits in *C201R/+* mice are not improved by over-expression of *GARS*. The severity of motor dysfunction in *C201R/+* mice was quantified using a wire hang test of grip strength. The mice were placed on a wire grid, which was then inverted and the latency to fall was determined. The test was stopped at 60 seconds. Each mouse was evaluated in three trials with a 30 sec inter-trial interval. The means ± SD of the trials are plotted, and each point represents an individual animal. This test was performed at 3 weeks (A, C) and 6 weeks (B, D). Some wild-type mice were noncompliant and failed to hang for the full 60 seconds. Mutants were never able to finish the task. Neither transgene improved the behavioral deficits in *C201R/+* mice at 3 or 6 weeks of age. (E, F) Nerve conduction velocities (NCVs) were also significantly reduced in this milder CMT2D mouse model and this was not improved by either transgene. NCVs were measured at 12 weeks for mice crossed with transgene A and at 8 weeks for mice crossed with transgene D. (E) In the cross between *C201R/+* mutant mice and transgene A the NCVs were: 36.1±5.4 for wild type (n = 6), 32.9±4.9 for WT; *TgA* (n = 6), 21.1±9.4 for *C201R/+* (n = 5), and 20.7±3.8 for *C201R/+*; *TgA* mice (n = 5). (F) In the cross between *C201R/+* mutant mice and transgene D, the NCVs were: 38.9±3.5 for wild type (n = 2), 33.9±7.3 for WT; *TgD* (n = 4), 17.3±1.9 for *C201R/+* (n = 5), and 22.6±2.5 for *C201R/+*; *TgD* mice (n = 6). Transgenes A and D did not significantly improve NCVs in *C201R/+* mutant mice.

To further substantiate these findings, we also tested the effects of both *TgA* and *TgD* in a second pathological *Gars* allele. The milder CMT2D disease model, *C201R/+*, has less severe overt neuromuscular phenotypes than the *Nmf249/+* mice. In this model, behavioral measures of peripheral nerve dysfunction are useful to detect the subtler phenotype. One behavioral measure is a wire hang test of grip strength. Mice were placed on a wire grid that was then inverted and the time that mice were able to suspend themselves was recorded. At 3 and 6 weeks of age, wild-type mice are often able to hang for a full minute, when the test is stopped. Neither transgene significantly improved performance on this test at either age tested (Figure 5A–5D).

To look for subclinical improvements in motor unit function, we tested nerve conduction on mice from this cross as well. Nerve conduction in *C201R/+* mice is significantly reduced to approximately 55% of wild-type levels. Neither TgA nor TgD significantly improved NCVs in the *C201R/+* mice (Figure 5E, 5F).

In summary, there were trends towards slight and variable improvements in body weight, axon diameter, and NCV in CMT2D mouse models when wild-type *GARS* is ubiquitously over-expressed, but none of these improvements were statistically significant, and the mice still have compromised motor function assessed by behavioral, histological, and electrophysiological measures compared to control animals.

### Wild-type *GARS* transgenes functionally substitute for an endogenous wild-type *Gars* allele

Although there was a robust increase in wild-type GARS protein detected by immunoblotting in both *TgA* and *TgD*, and animals with the transgenes had significantly higher tRNA^Gly^ charging activity, it remained to be demonstrated that the transgene products in these mice can substitute for a loss of wild-type *Gars*. Human *GARS* can functionally replace the fruit fly ortholog, *Aats-gly* [28], but does not rescue yeast lacking *GRS1* (A.A. unpublished data). To confirm that our human *GARS* transgene could replace mouse *Gars*, we tested whether the transgenes could restore wild-type function in place of the *XM256* loss-of-function gene trap allele [25]. The *XM256* allele unambiguously causes a loss-of-function by intercepting splicing and reducing mRNA levels of *Gars* assayed by northern blot, as well as reducing enzymatic activity in tissue homogenates from heterozygous mice [25].

Compound heterozygotes of the gene trap allele with either mutant allele (*C201R/XM256* and *Nmf249/XM256*) die embryonically [25], [26]. We crossed *XM256/+*; *TgA* or *TgD* mice with *C201R/+* mice, and the offspring included both viable *C201R/XM256*; *TgA* and *C201R/XM256*; *TgD* mice. This shows that both *TgA* and *TgD* were able to restore viability by substituting for the endogenous wild-type *Gars* allele. We expected *C201R/XM256*; *Tg* mice to have a mild neuropathy because of the pathogenic *C201R* allele, and “full rescue” would therefore constitute viable mice with a phenotype comparable to *C201R/+*.

Chi-square analysis showed that *C201R/XM256*; *TgA* and *C201R/XM256*; *TgD* mice were born at expected Mendelian ratios (6/48, p = 1.0, and 4/63, p = 0.14, respectively), while *C201R/XM256* mice without the transgenes were not viable, as expected, and represented a significantly absent class (p = 0.009, p = 0.003, respectively).

**Figure 6:**
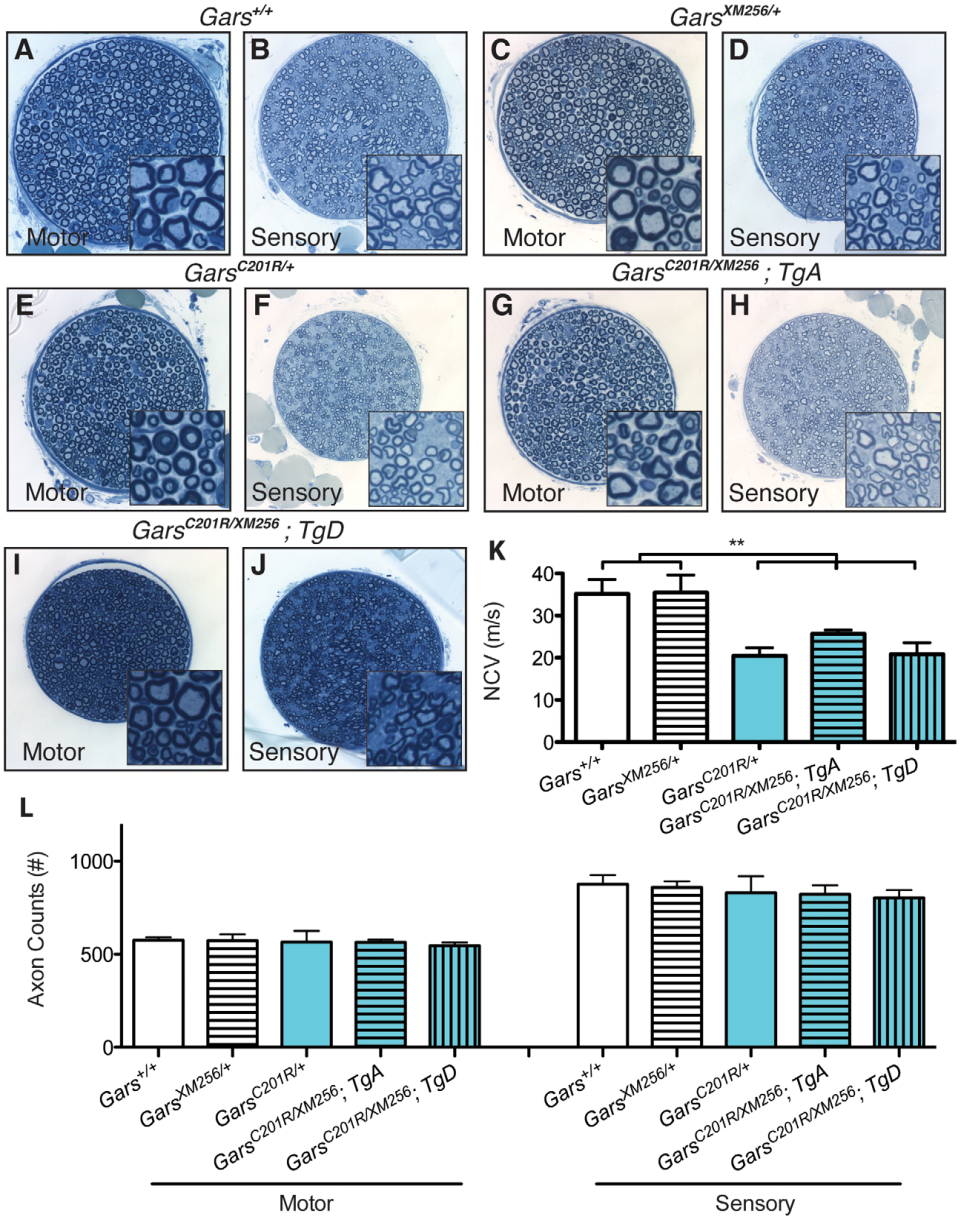
Transgenes A and D restore viability to *C201R/XM256* mice. Motor and sensory branches of the femoral nerves of rescued *C201R/XM256; Tg* mice were isolated at 8 weeks of age and compared to littermate wild-type, *XM256/+*, and *C201R/+* control mice. Toluidine blue stained sections show that motor and sensory nerves from wild type (A, B) and *XM256/+* mice (C, D) control mice are qualitatively similar to nerves from *C201R/+* (E, F), *C201R/XM256; TgA* (G, H), and *C201R/XM256; TgD* (I, J) mice. (K) Functionally, nerve conduction velocities in wild type and *XM256/+* are not significantly different (35.2±3.4 (n = 7), and 35.5±4.2 (n = 4), respectively). However, as anticipated, values are reduced in *C201R/+* mice (20.5±1.9 (n = 6), p<0.001). Conduction velocities in rescued *C201R/XM256* mice were not significantly different than *C201R/+* mice, but still significantly reduced compared to wild type controls (p<0.001). Values were: *C201R/XM256*;*TgA* = 25.7±0.9 (n = 4) and *C201R/XM256*;*TgD* = 20.9±2.7 (n = 3). (L) Axon numbers in the motor branch of the femoral nerve were as follows: wild type = 575±17 (n = 17), *XM256/+* = 573±34 (n = 6), *C201R/+* = 566±59 (n = 6), *C201R/XM256;TgA* = 564±14 (n = 6), *C201R/XM256;TgD* = 546±17 (n = 4). There were no significant differences in these values. Axon numbers in the sensory branch of the femoral nerve were also unchanged between genotypes as follows: wild type = 876±50, *XM256/+* = 859±32, *C201R/+* = 830±89, *C201R/XM256;TgA* = 822±49, *C201R/XM256;TgD* = 803±42. Animal numbers are the same as for motor axons except for wild type, where n = 7.

In addition to restoration of viability, the *C201R/XM256* mice with the transgenes were restored to a *C201R/+*-like neuropathy. The rescued mice had femoral nerve pathology and axon numbers that were not significantly different from *C201R/+* mice or controls at eight weeks of age (Figure 6A–6J, 6L). As previously reported, neither the *XM256/+* nor the *C201R/+* mice had a decrease in myelinated axon number in the motor or sensory branch of the femoral nerve, whereas the *Nmf249/+* mice do have reduced axon numbers (Figure 6 and [25], [26]). Therefore, the rescued mice do not show an increased severity in their neuropathy by this measure. We also tested this functionally by measuring nerve conduction velocities, which are normal in *XM256/+* mice (Figure 6K), but are significantly reduced in *C201R/+* mice (Figure 5E and 5F, Figure 6K). Conduction velocities in rescued mice were significantly below control values, and were not significantly different than *C201R/+* values.

**Figure 7:**
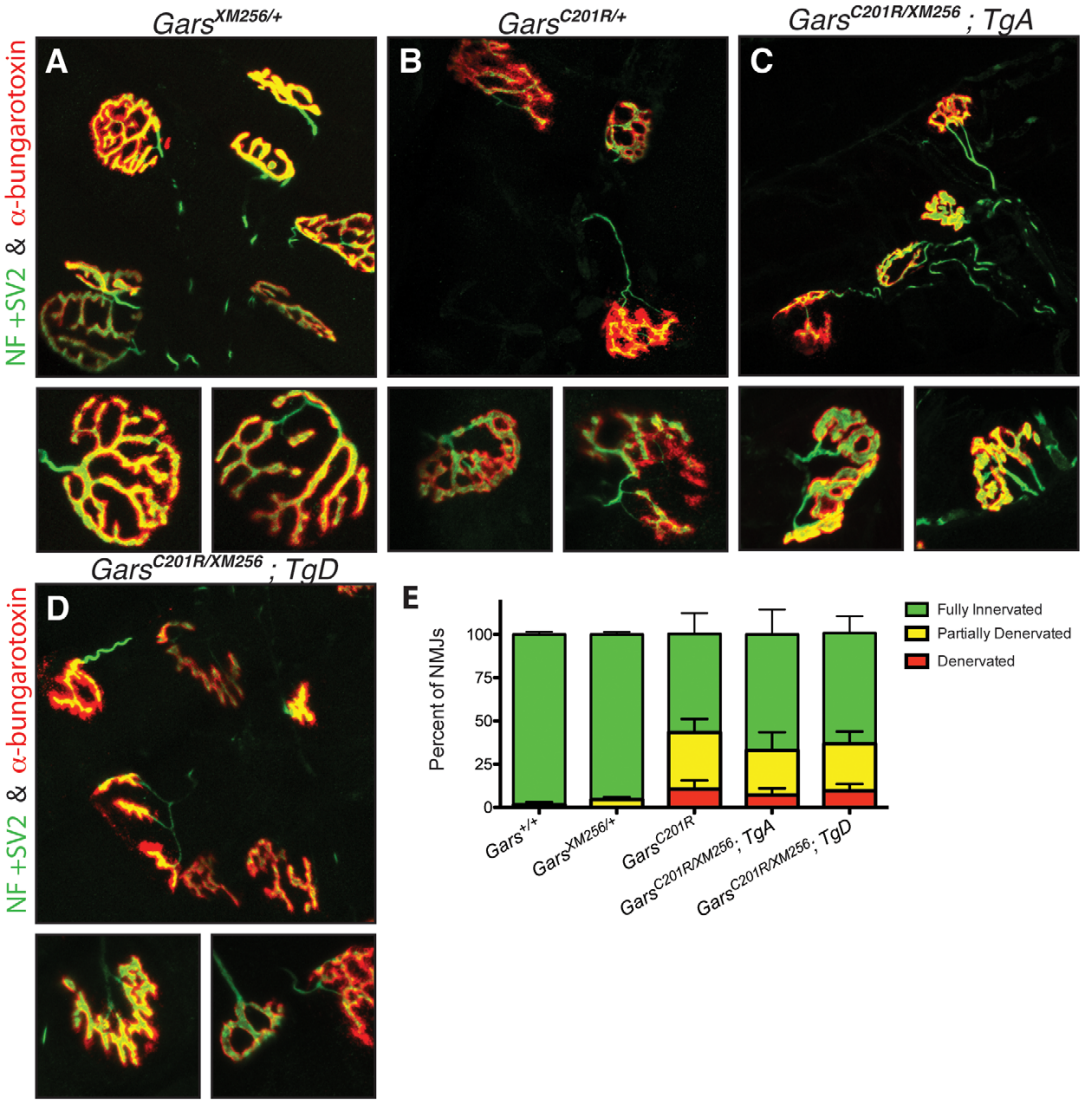
Neuromuscular junctions in rescued mice are similar to those in *C201R/+* mice. Neuromuscular junctions (NMJs) in wild-type (not shown) and *XM256/+* (A) mice are mature pretzel-like structures. B) While most NMJs in *C201R/+* mice are fully innervated, some NMJs are partially denervated or fully denervated. In rescued *C201R/XM256; TgA* (C), and *C201R/XM256; TgD* (D) mice, NMJ morphology is similar to that of *C201R/+*mice, with regions of denervation in some NMJs with neighboring NMJs that are fully innervated. NMJ occupancy was evaluated in these mice to quantitatively determine the severity of the phenotype. (E) 98.5±1.5% (n = 6) of NMJs were fully occupied in wild-type mice and 95.5±1.4% (n = 6) in *XM256/+* mice. These were both significantly higher than the 57.0±12.0% (n = 6) of NMJs that were fully occupied in *C201R/+* mice (p<0.001). The percentage of fully innervated NMJs was 67.0±14.3% (n = 6) in rescued *C201R/XM256; TgA* mice and 64.0±9.8% (n = 4) in *C201R/XM256; TgD* mice, and was not signficantly different from *C201R/+* mice.

To further verify that the transgenes fully rescued the neuropathy to the severity anticipated for *C201R/+*, we also examined NMJ morphology and occupancy at 8 weeks of age. The NMJs of *XM256/+* mice are indistinguishable in morphology and occupancy from wild-type mice (Figure 7A and not shown), and form complex pretzel structures with complete overlap between pre and post-synaptic markers. NMJs in *C201R/+* mice (Figure 7B) have regions of immature morphology or denervation that are noticeably different from wild-type, but are not as dysmorphic as the NMJs in *Nmf249/+* mice. *C201R/XM256* mice with *TgA* or *TgD* had neuromuscular junction morphology similar to that of *C201R/+* mice (Figure 7C, 7D). No significant difference in the proportion of NMJs that were fully occupied was observed between *C201R/+*, *C201R/XM256*; *TgA*, and *C201R/XM256*; *TgD* mice (Figure 7E).

While the severity of the neuropathy was the same in *C201R/+* and *C201R/XM256*; *Tg* mice, the rescued mice had significantly lower body weights than wild type or *XM256/+* animals (Figure S3A). The muscle weight/body weight ratio was not reduced in these mice, suggesting that the lower body weight was not a result of neuropathic muscle degeneration (Figure S3B). Therefore, the reduced body weight probably reflects some insufficiency in transgene expression in other tissues. However, the viability of compound heterozygous *C201R/XM256* mice with *TgA* and *TgD* indicates that both produce functional protein and both are capable of substituting for a wild type allele of *Gars*.

### Increasing the genetic dosage of mutant *Gars* increases the severity of neuropathy

To examine the effects of increasing the genetic dose of the mutant alleles of *Gars* and to further parse if there is a loss-of-function component to the amino acid substitutions, we generated *C201R* homozygotes and *C201R/Nmf249* compound heterozygotes, both with and without *TgD*.

*C201R* homozygous mice have a more severe neuropathy phenotype than *C201R/+* heterozygotes, and typically die at about two weeks of age [26]. It is unclear whether the more severe phenotype in homozygotes is due to a double-dose of a toxic mutant protein, to a compounded loss-of-function, or a combination of these effects. Based on the ability of *TgD* to substitute for a wild type *Gars* allele in rescuing viability in combination with *XM256*, we hypothesized that a loss of function would be corrected by the presence of the transgene, whereas increased toxicity from the higher dose of mutant protein would not be corrected, based on the inability of the transgene to improve the neuropathy phenotype in *C201R/+* mice.

**Figure 8:**
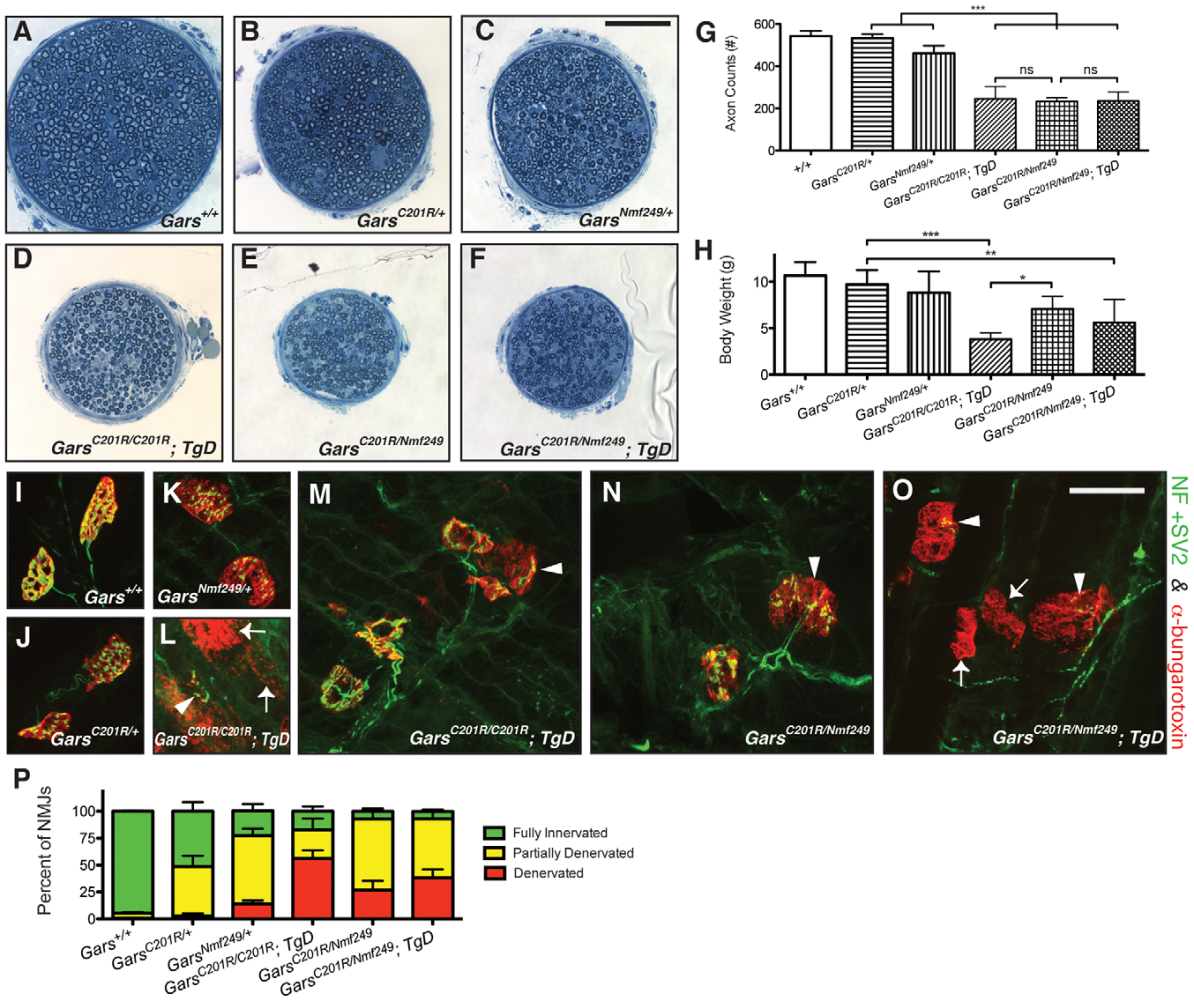
Increased dosage of mutant *Gars* increases the severity of neuropathy, but wild-type over-expression only improves viability in homozygous *C201R* mice. The motor branch of the femoral nerve was used to assess the severity of the neuropathy in homozygous and compound heterozygous mice at post-natal day 17. Motor nerves from wild-type (A), *C201R/+*(B), and *Nmf249/+* (C), were compared to nerves from *C201R/C201R*; *TgD* (D), *C201R/Nmf249* (E), and *C201R/Nmf249*; *TgD* (F), which were smaller. The scale bar (shown in C) is 50 µm. The gross observation that the motor nerves are smaller in the homozygous and compound heterozygous mutant mice was confirmed quantitatively. (G) Motor nerve count averages were as follows: 543±24 for wild type mice (n = 8), 533±20 for *C201R/+* mice (n = 7), 461±36 for *Nmf249/+* mice (n = 5), 246±58 for *C201R/C201R*; *TgD* mice (n = 9), 233±18 for *C201R/Nmf249* mice (n = 4), and 236±42 for *C201R/Nmf249*; *TgD* mice (n = 5). *C201R/C201R*; *TgD*, *C201R/Nmf249*, and *C201R/Nmf249*; *TgD* mice had fewer motor axons than *Nmf249/+* mice (p<0.001 for all three genotypes). There were no significant differences among the three genotypes that are homozygous or compound heterozygous for *Gars* mutation. (H) Body weights were as follows: wild type = 10.7±1.4 g (n = 7), *C201R/+* = 9.7±1.5 (n = 8), *Nmf249/+* = 8.8±2.3 (n = 5), *C201R/C201R*; *TgD* = 3.8±0.7 (n = 9), *C201R/Nmf249*  =  7.1±1.4 (n = 4), and *C201R/Nmf249*; *TgD* = 5.6±2.5 (n = 5). *C201R/C201R*; *TgD*, and *C201R/Nmf249*; *TgD* mice weighed less than *C201R/+* mice (p<0.001, p<0.01, respectively). *C201R/Nmf249* mice weighed more than *C201R/C201R*; *TgD* mice (p<0.05) and were not significantly different from *C201R/+* mice. Plantaris muscles were removed from P17 pups for neuromuscular junction imaging (I-O). Green pre-synaptic staining is from NF and SV2 antibodies, red post-synaptic staining is α-bungarotoxin. Nearly mature NMJs were seen in wild-type animals (I). As shown in Figure 3 and Figure 7, *C201R/+* (J) and *Nmf249/+* (K) have abnormal NMJ morphology with areas of denervation in some NMJs and others that are fully denervated. More severe signs of denervation were seen in *C201R/C201R*; *TgD*, *C201R/Nmf249*, and *C201R/Nmf249*; *TgD* mice. There was a wide range of dysmorphic NMJ pathology in *C201R/C201R*; *TgD* mice. Some sections had mostly denervated NMJs (arrows) (L), while other areas had some partially innervated junctions (arrowheads) (M). A similar mix of morphologies was seen with *C201R/Nmf249* mice (N) and *C201R/Nmf249*; *TgD* mice (O). The scale of I-O is indicated by the 28 µm bar in O. Scoring of neuromuscular junctions was performed to quantitatively compare the proportion of NMJs that were fully innervated (green), partially innervated (yellow), and denervated (red) (P). In *Nmf249/+* mice, 14.2±3.0% of NMJs were denervated. Significantly more junctions were fully denervated in *C201R/C201R*; *TgD* mice where 56.2±7.5% of NMJs were denervated, and in and *C201R/Nmf249*; *TgD* mice where 38.2±8.0% of NMJs were denervated (p<0.001 for both comparisons). Compared to *C201R/+* mice, where 2.8±2.4% of NMJs were denervated, significantly more were denervated in *C201R/Nmf249* mice where 26.8±8.7% of junctions were denervated, but this was not statistically different from the *Nmf249/+* mice. Compared to both *C201R/Nmf249* and *C201R/Nmf249*; *TgD* mice, *C201R/C201R*; *TgD* mice had a greater proportion of denervated NMJs (p<0.05, p<0.001, respectively).

Consistent with previous reports, we found that *C201R/C201R* mice were subviable (X^2^ p = 0.016) [26]. Only two homozygous *C201R* mice were recovered in 69 offspring (1/8 anticipated), and these died by P12 before they could be analyzed. However, *C201R/C201R; TgD* mice were born at expected Mendelian ratios (11/69, X^2^ p = 0.39), and consistently lived to post-natal day 17, when tissues were collected and analyzed. The maximum observed lifespan for these mice was to P20. Despite the restoration of wild-type GARS function with *TgD*, these mice had a more severe neuropathy than *C201R/+* heterozygotes. The *C201R/C201R;TgD* mice had approximately a 50% reduction in axon numbers in the motor branch of the femoral nerve (Figure 8A, 8B, 8D, 8G), and the mice were <5 grams in weight at P17 (Figure 8H). Neuromuscular junction defects were similarly increased in severity (Figure 8I, 8J, 8L, 8M, 8P). At P17, wild-type NMJs have developed into mature pretzel shapes, but *C201R/+* NMJs are arrested at a less-mature, plaque-like morphology, and show an increased incidence of partially innervated junctions. The *C201R/C201R;TgD* mice demonstrated frank denervation at almost half of the postsynaptic sites. In agreement with this, we could not obtain nerve conduction velocities from these mice, because muscle action potentials were too small to reliably record (n = 8). Thus, the reduced viability embryonically and in neonates seen in *C201R* homozygous mice was corrected by the *TgD*, as demonstrated by the recovery of *C201R/C201R;TgD* mice at the expected Mendelian ratios and their survival to P17. However, the increased genetic dosage of the *C201R* allele leads to a more severe neuropathy.

To further examine this effect of genetic dosage and apparent recessive partial-loss-of-function, we also made compound heterozygous *C201R/Nmf249* mice. Interestingly, both *C201R/Nmf249* and *C201R/Nmf249*; *TgD* mice were viable and born at expected Mendelian frequencies (5/33, X^2^ p = 0.95 and 4/33, p = 0.65, respectively). Considering that both *C201R/XM256* and *Nmf249/XM256* are nonviable in the absence of a wild-type transgene, the viability of *C201R/Nmf249* mice suggests that there is intragenic complementation between the two mutant alleles that alleviates the loss-of-function and subsequent reduction in viability at birth.

Like homozygous *C201R/C201R*; *TgD* mice, the motor branch of the femoral nerve is noticeably smaller and axon numbers are significantly reduced in *C201R/Nmf249* and *C201R/Nmf249*; *TgD* mice compared to wild-type or heterozygous littermate controls (Figure 8A–8G). No significant difference in axon numbers was seen between the *C201R/C201R*; *TgD*, *C201R/Nmf249*, and *C201R/Nmf249*; *TgD* genotypes. The same qualitative and quantitative differences were seen in the sensory nerves of these animals (Figure S4).

The overall health and overt appearance of the compound heterozygous mice was better than that of homozygous *C201R/C201R;TgD* mice. The body weights of *C201R/Nmf249* and *C201R/Nmf249*; *TgD* mice were significantly lower than *C201R/+* mice (Figure 8H), and *C201R/C201R*; *TgD* weighed significantly less than *C201R/Nmf249* mice.

Morphological changes seen in the neuromuscular junctions of *C201R/Nmf249* and *C201R/Nmf249*; *TgD* mice were also more severe than either allele as a heterozygote and reflected the axon loss seen in the femoral nerves. Like *C201R/+* mice at P17, *Nmf249/+* mice have partially denervated NMJs (Figure 8K) with areas of disorganized post-synaptic staining, but also more examples of complete denervation. Like the *C201R/C210R;TgD* mice, compound heterozygotes with or without *TgD* had even more dysmorphic NMJ morphology than *Nmf249/+* heterozygotes, with a majority of NMJs that were partially or fully denervated and very few that were fully innervated (Figure 8N–8P). There were significantly more denervated NMJs in *C201R/C201R*; *TgD*, *C201R/Nmf249*, and *C201R/Nmf249*; *TgD* mice compared to *Nmf249/+* mice (Figure 8P). Compound muscle action potentials were again too small to reliably record in *C201R/Nmf249* (n = 4), and *C201R/Nmf249*; *TgD* (n = 5) mice.

Therefore, the presence of *TgD* did not alter viability, axon number, body weight, or NMJ occupancy of the compound heterozygous mice, consistent with intragenic complementation improving the recessive loss-of-function sub-viability phenotype observed in *C201R* homozygotes. In both *C201R/Nmf249* compound heterozygotes and *C201R* homozygotes, the severity of the neuropathy increases as the genetic dosage of the mutant *Gars* increases, even in the presence of the wild-type transgene.

## Discussion

To summarize our results, the over-expression of functional, wild-type *GARS* does not suppress the neuropathy caused by dominant amino acid substitutions, but does rescue reduced viability seen with homozygous point mutations or in point mutations combined with a null allele. Furthermore, the neuropathy is worsened with homozygous or compound heterozygous amino acid substitution alleles (Table 1). From these results we conclude that the dominant mechanism underlying *GARS* mutations and the degeneration of peripheral axons is a toxic gain of function by the mutant GARS protein. This toxicity depends on the dosage of the mutant protein and is not mitigated or out-competed by the over-expression of wild-type *GARS*, suggesting that the neuropathy is not likely to be caused by a loss-of-function or a simple dominant-negative mechanism. There is also a loss of GARS function with the mutations, but this is only seen when alleles with amino-acid changes are made homozygous or placed over a loss-of-function allele created by a gene trap insertion. The recessive loss of function reduces viability, but is corrected by the transgenic over-expression of wild-type *GARS*, leaving these mice with only the dose-dependent neuropathy caused by expression of the mutant protein. Interestingly, compound heterozygous mice carrying two different alleles with pathological amino-acid changes show “additive” neuropathy that is more severe than either allele alone as a heterozygote, and is comparable in severity to a point mutation as a homozygote. However, compound heterozygous mice do not show reduced neonatal viability associated with a loss of function, and the phenotype is not improved by over-expression of wild-type *GARS*, indicating that there is intragenic complementation and little or no loss of function in the compound heterozygotes. The basis for these conclusions and the implications for CMT are discussed below.

**Table 1:** A summary of the genotypes of mice used in these studies indicating the viability and neuropathy phenotypes associated with each genotype.

Genotype	Viability	Neuropathy
*Gars^+/+^*	normal	−
*Gars^+/+^; TgA*	normal	−
*Gars^+/+^; TgD*	normal	−
*Gars^Nmf249/+^*	normal	+++
*Gars^Nmf249/+^; TgA*	normal	+++
*Gars^Nmf249/+^; TgD*	normal	+++
*Gars^C201R/+^*	normal	++
*Gars^C201R/+^; TgA*	normal	++
*Gars^C201R/+^; TgD*	normal	++
*Gars^XM256/+^*	normal	−
*Gars^C201R/XM256^*	embryonic lethal	ND
*Gars^C201R/XM256^; TgA*	normal	++
*Gars^C201R/XM256^; TgD*	normal	++
*Gars^C201R/C201R^*	reduced viability	ND
*Gars^C201R/C201R^; TgD*	normal viability, reduced lifespan (P17)	++++
*Gars^C201R/Nmf249^*	normal viability, reduced lifespan	++++
*Gars^C201R/Nmf249^; TgD*	normal viability, reduced lifespan	++++

KEY: ND none determined, − normal phenotype, ++ moderate, +++ severe, ++++ very severe.

### Peripheral neuropathy phenotype is caused by a gain of function

Overall, no significant improvement in axonopathy phenotypes was seen in CMT2D model mice over-expressing wild-type GARS. Two transgenes with similar levels of expression were used in these crosses and yielded effectively the same results. Neither transgene prevented axon degeneration or improved nerve conduction velocity or NMJ morphology and occupancy in the *Nmf249/+* mice at one month of age. The only positive effect of the transgenes was a minor improvement in axon diameter. Similarly, neither transgene improved behavioral measures at three or six weeks of age, or NCVs at eight or twelve weeks of age in *C201R/+* mice. Thus, over-expression of wild-type *GARS* did not suppress the dominant neuropathy phenotype. It is also notable that the over-expression of wild-type *GARS* did not cause any phenotype on its own in either transgenic line.

These crosses, and the observation that *XM256/+* mice do not have a neuropathy, show that the mutant protein itself is toxic and causes peripheral nerve degeneration. The toxicity can be intensified, and the neuropathy worsened, by increasing the dose of mutant protein in either homozygotes or compound heterozygotes. Lifespan, NMJ occupancy, axon numbers and body weight are significantly reduced in *C201R/Nmf249*, *C201R/Nmf249*; *TgD*, and *C201R/C201R*; *TgD* mice below the levels of *Nmf249/+* and *C201R/+* mice, which only have one copy of the mutant protein. This is consistent with experiments with *YARS* in *Drosophila*, which also indicate that the mutant tRNA synthetase has a dose-dependent toxicity [29].

### Loss-of-function component is responsible for reduced viability

Although the peripheral axon degeneration is caused by toxicity of the mutant protein, the mutations do have loss-of-function characteristics. Homozygous *C201R* mice are subviable, and neither allele is viable in combination with the gene trap allele, despite the *Nmf249/+* P278KY protein being fully active in cell-free activity assays of amino acylation and tRNA charging [25].

The embryonic lethality observed in these *Gars* alleles is probably not related to the neuropathy phenotype. In fact, a functioning nervous system is not required during embryonic development. Mice deficient for proteins that are crucial for the function of the central and peripheral nervous system are born in the anticipated numbers, but do not survive independently after birth [30]–[32]. GARS is necessary for translation in every cell, and loss of function in non-neurological tissue is a likely cause of the embryonic lethality.

With transgenic over-expression of wild-type protein, we were able to restore full viability to *C201R/XM256* mice, which otherwise die embryonically, and *C201R/C201R* mice, which are otherwise subviable. It is notable that we were unable to rescue *XM256* as a homozygote with either transgene. This may be due to a closely linked second mutation on the gene trap chromosome, but is more likely due to an insufficiency of transgene expression at some critical point in development. This is supported by the reduced body weight of *XM256/C201R* mice with transgene rescue despite full rescue of viability and neurological function equivalent to a wild type allele. Presumably, the *C201R* allele provides sufficient GARS activity in whatever tissue may be lacking transgene expression, allowing rescue of the point mutation over the gene trap. Thus, over-expression of wild-type *GARS* can rescue postnatal viability, but does not improve the neuropathic phenotype, suggesting the loss of viability is caused by a loss of function for which the transgene can compensate, whereas the neuropathy is a gain of function that the transgene cannot correct.

### Intragenic complementation confirms that toxicity is the major determinant of disease severity

*C201R* homozygous mice have decreased viability, and when they are born they have a severe neuropathy. We expected *C201R/Nmf249* mice to be at least as severe because the *Nmf249* allele is more pathogenic than the *C201R* allele despite its normal enzymatic activity. Surprisingly, the compound heterozygous mice were as healthy as or even healthier than *C201R* homozygotes with *TgD*. The *C201R/Nmf249* mice were fully viable and weighed significantly more than *C201R/C201R*; *TgD* mice, although their neuropathy was markedly more severe than either allele as a heterozygote. Therefore, the *C201R* and *Nmf249* alleles are capable of intragenic complementation in regard to the loss-of-function phenotype, but still display “additive” severity for the gain-of-function neuropathy phenotype. Over-expression of wild-type *GARS* did not improve the phenotype in the compound heterozygous mice, which are severely affected because they express two mutant versions of the protein. This suggests that the loss-of-function aspect of the *C201R* homozygotes that is rescued by the transgene is not a factor in the compound heterozygotes, and that the dose of the mutant protein determines the severity of the neuropathy. It also suggests that this loss of function, perhaps in enzyme charging function, differs between the alleles, because *C201R/Nmf249* mice are more viable than *C201R/C201R* mice even though the *Nmf249* allele is more neurotoxic than the *C201R* allele.

### Therapies for tRNA–synthetase–linked neuropathies should selectively target the mutant allele

Dominant mutations in *GARS*, *YARS*, and *AARS* all cause CMT [14]–[16]. Our results suggest that mutant forms of GARS adopt a pathological function that may be impacting the normal role of GARS in translation, but that the loss-of-function effects are recessive, and would not be an important factor in CMT patients with dominantly inherited disease. Instead, the neuropathy is a caused by a gain of function, demonstrated by the inability of functional wild-type transgenic *GARS* to rescue the phenotype either by restoring lost activity or outcompeting this pathological function. One patient reported to carry compound heterozygous mutations in *KARS* may be the exception to this, but it is notable that this genotype was associated with peripheral neuropathy with additional neurological and non-neurological symptoms [17].

The interplay between normal protein function and pathogenic function has been dissected in other neurodegenerative diseases. For example, hereditary sensory and autonomic neuropathy (HSAN) type 1A is caused by mutations in the serine palmitoyl transferase gene (*SPTLC1*), which alter the protein's amino acid substrate specificity so that the mutant produces two neurotoxic atypical deoxysphingoid bases [33]–[35]. Over-expression of wild-type SPTLC1 rescued a transgenic mouse model of the disease by outcompeting mutant forms of SPTLC1 in heterodimer formation with SPTLC2, restoring the substrate specificity, lowering levels of neurotoxic sphingoid bases, and demonstrating that mutant SPTLC1 is not inherently toxic [36]. Our work suggests that mutant GARS protein is toxic independent of wild-type protein levels. These results are more like what has been observed in SOD1-linked amyotrophic lateral sclerosis, which is caused by dominant mutations and is not improved with wild-type over-expression [37].

These conclusions have implications for therapeutic approaches. To reduce the effect of mutant GARS, the expression of the mutant allele must be diminished. Future therapeutic approaches should focus on allele specific knockdown of the mutant gene expression; alternatively, the specific pathways affected by mutant GARS toxicity could be identified and targeted.

## Materials and Methods

### Generation of transgenic mice

The Ins-CMV-C-B-A vector [38] was modified to include a Gateway conversion cassette (Invitrogen) in the multiple cloning site, which includes a ccdB cassette flanked by attR1 and attR2 recombination sequences. A Gateway LR clonase reaction was used to insert a PCR amplicon of the GARS cDNA (human) into the pINS2-Gateway vector. The cDNA amplicon that was inserted into pINS2 included 47 basepairs of the endogenous GARS 5′ UTR upstream of the start site of the mitochondrial isoform, and 19 basepairs of endogenous 3′UTR flanked by 5′ and 3′ untranslated regions (UTR) from rabbit β globin also containing a polyadenylation signal at the 3′ end. The cDNA and control elements are flanked by two chicken β-globin 5′HS4 insulators, which have been shown to reduce variability in transgene expression caused by position effects of insertion site [39]. Pronuclear injection of this construct into single cell FVB/N embryos was performed in the NHGRI Transgenic Mouse Core according to existing protocols [40].

### Mouse strains, husbandry, and genotyping

All mouse husbandry and procedures were conducted according to the NIH Guide for Care and Use of Laboratory Animals and were approved by NINDS or The Jackson Laboratory Animal Care and Use Committee. Tail, toe or ear tissue was lysed with proteinase K incubation at 55°C and boiled for 10 min to inactivate the proteinase K. DNA derived from the mouse tissue was then used in PCR to determine genotype.

Primers GARS WT Tg F (5′-CCCATTACTGGAAATGATCTA-3′) and GARS WT Tg R (5′-TTTCCGAGCGGACTGTCCGC-3′), which anneal to exons 7 and 9, respectively, were used to determine if the wild-type transgene was present. Primers targeting *Gars* intron 6F (5′-GCCTTGTTCTGTAACGTTTGCAC-3′) and a primer specific to the *Nmf249* allele (5′-CCAGGCATATTTCCTCCATATTT-3′) were used to identify mice with the *Nmf249* mutation [25]. Primers Gars C201R F (5′-CACGTGCTTGCTCTAGCAAGA-3′) and Gars C201R R (5′-GTCTACCACTGAACACAGTCC-3′) were used in PCR reaction, and the product was digested with *HhaI* restriction enzyme. PCR products containing the pathogenic mutation are digested into two smaller bands [26]. Primers BgeoR (5′-CGCCAGGGTTTTCCCAGT-3′) and En2-i1F (5′-AATGCCCAACACTTGTATGG-3′) were used to determine if the *XM256* gene trap allele was present. To screen for *XM256/XM256* mice, primers flanking the gene trap insertion 519 bp into intron 2 of *Gars*, GarsXMvsWT2F (5′-GCTTCCGCACTACCTGAACCCAAACT-3′) and GarsXMvsWT2R (5′-TGAATTCAGCAGCCCCCTCTGTACCC-3′), were used. No *XM256/XM256* mice were identified, with or without the transgene. PCR amplification indicated the wild-type allele was present. All genotyping products were resolved on 2% agarose gels with ethidium bromide (Sigma, St. Louis, MO). *Nmf249* mice are maintained on a C57BL/6 background and are occasionally outcrossed to CAST/Ei to improve their ability to breed [21]. *C201R* mice were originally on a mixed C57BL/6 and C3H background and subsequently bred into a C57BL/6 background. The *TgA* and *TgD* alleles were maintained on a pure FVB/N background with one exception: the *TgA* mice that were used in the cross with *C201R* mice were on a FVB/N X C57BL/6 F1 hybrid background. Therefore, experiments were done primarily in a [FVB/N X BL/6] F1 background unless two matings were required to generate homozygotes or compound heterozygotes, in which case mice were effectively in an [N2] C57BL/6 background. All control animals were siblings of the experimental genotypes.

### Tissue lysate preparation

Spinal cord and sciatic nerve were isolated from animals immediately after they were euthanized by CO_2_ inhalation. The tissues were frozen in liquid nitrogen and stored at −80°C. The tissues were then homogenized in 1% NP-40 in phosphate buffered saline (PBS) supplemented with Protease Inhibitor Cocktail Tablets (Roche, Basal, Switzerland) using a PowerGen Model 125 Homogenizer (Fisher Scientific, Pittsburgh, PA) then centrifuged at 14,000 g for 10 min at 4°C. Cleared homogenates were then sonicated at 4°C and centrifuged again at 14,000 g for 10 min. Protein concentrations were assessed using a Bradford assay (BioRad, Hurcules, CA). 20 µg of protein was then analyzed by immunoblot.

### Western blots

Protein lysates were resolved on Novex 10% Tris-Glycine Gels (Invitrogen, Carlsbad, CA) and transferred to an Invitrolon PVDF membrane for western blot analysis. Membranes were blocked with 5% skim milk in TBST (1× Tris-buffered saline, 0.1% Tween-20), and incubated overnight with GARS rabbit polyclonal antibody ab42905 (1∶2,000) (Abcam, Cambridge, MA) and β actin mouse monoclonal antibody clone AC-74 (1∶10,000)(Sigma Aldrich, St. Louis, MO) diluted in blocking solution at 4° C. Following three 10 min washes in TBST, the blots were incubated with the appropriate horseradish peroxidase-conjugated secondary antibodies (Jackson ImmunoResearch, West Grove, PA) diluted in blocking solution. After three 10-min washes in TBST, the blots were developed using Western Lightening Plus-ECL, Enhanced Chemiluminescence Substrate (Perkin Elmer, Waltham, MA).

### Teased nerve immunohistochemistry

Teased nerve fibers were stained as described in the methods of [21], [41]. In brief, sciatic nerves were excised, immediately placed in fresh 4% paraformaldehyde, and fixed on ice for 15 minutes. Nerves were then transferred to ice cold PBS for dissection and teasing. Using #5 forceps and 30 gauge needles, nerve sheaths were removed and the nerves were cut into 1 cm segments and teased apart at one end. The nerve segments were then transferred to a fresh Superfrost Plus Gold slides (Fisher Scientific, Pittsburgh, PA) and pulled from a drop of PBS onto a dry section of the slide to straighten the fibers for imaging. The slides were then dried overnight at room temperature and incubated in acetone at −20°C for 10 min. The samples were then rehydrated with two 5 min incubations in PBS and blocked with 5% normal goat serum in PBS with 0.5% Triton X-100 for 1 h at room temperature. Primary antibodies rabbit anti-GARS (1∶500) (Abcam), mouse anti-neurofilament with the 2H3 antibody (1∶500)(Developmental Studies Hybridoma Bank, Iowa City, IA) were gently placed on the slide, covered with Parafilm coverslips (Pechinery Plastic, Chicago, IL) and stored in a humidified chamber overnight at 4° Celsius. After three 5-min washes in PBS, the samples were incubated in the following secondary antibodies diluted 1∶1,000 in blocking solution: AlexaFluor 555 goat anti-rabbit, and AlexaFluor 488 goat anti-mouse IgG_1_ (y1) (Invitrogen, Carlsbad, CA). The samples were covered with Parafilm coverslips and incubated for 2 h at room temperature. The samples were then washed three times in PBS for 5 min each in watch glasses and mounted with Vectashield (Vector Labs, Burlingame, CA).

### Aminoacylation assays

Aminoacylation assays were performed at room temperature in a reaction mixture containing 50 mM HEPES (pH 7.5), 20 mM KCl, 2 mM ATP, 5 mM MgCl_2_, 1 mM DTT, 19 µM L-glycine (or L-alanine), 1 µM ^3^H-L-glycine (or ^3^H-L-alanine), 2 µM transcribed human tRNA^Gly^_CCC_ [for GlyRS activity] or 120 µM yeast total tRNA (Roche Diagnostics, Indianapolis, IN) [for AlaRS activity] and 30 µg total protein from tissue homogenates. Reactions were initiated by addition of reaction mixture and tRNA to tissue homogenates. Aliquots were quenched at different time points and precipitated in 96-well Multiscreen filter plates (Millipore) as described previously [42]. After washing and elution by NaOH, samples were counted in a MicroBeta plate reader (PerkinElmer Life Sciences). Initial rates were measured from slopes obtained by fitting the data to a non-linear regression curve using GraphPad Prism 5 software. For TgA, 3 wild type and 4 transgenic littermates were sampled at 9 weeks of age: for TgD, 4 wild type and 3 transgenic littermates were tested at 6 weeks of age.

### Motor and sensory nerve analysis

The sensory and motor branches of the femoral nerve were isolated and fixed overnight in 2% glutaraldehyde and 2% paraformaldehyde in a 0.1 M cacodylate buffer. The tissue was then processed for transmission electron microscopy and embedded in plastic before 0.5 µm sections were cut and stained with toluidine blue. For more information see [43]. For axon counting and axon diameter measurement the images were captured using a Nikon Eclipse E600 microscope with 40× and 100× objectives. Axon counts were done using the Cell Counter Plug-in in ImageJ. Left and right nerves were averaged. Axon diameters were measured using the Measure and Label Plugin, also in ImageJ.

### NMJ imaging and analysis

Mouse plantaris muscles were surgically removed and fixed in freshly prepared 2% paraformaldehyde in PBS for four hours. The samples were then transferred to a blocking and permeabilizing solution of 5% normal goat serum and 0.5% Triton-X 100 in PBS for 1 h before they were pressed between two glass slides using a binder clip for 15 min, after which they were returned to the blocking and permeabilizing solution. The samples were then incubated overnight at 4°C with 1∶1,000 dilutions of anti-SV2 and anti-neurofilament (2H3) primary antibodies (Developmental Studies Hybridoma Bank, Iowa City, IA). After at least three 1 h washes in PBS with 0.5% Triton-X 100, the samples were transferred to blocking and permeabilizing solution with AlexaFluor 488 goat anti-mouse IgG_1_ (y1) (Invitrogen, Carlsbad, CA) and α-bungarotoxin conjugated with Alexa Fluor 594. After incubation overnight at 4°C, the samples were washed three times for 1 h each and mounted with Vectashield mounting media (Vector Labs, Burlingame, CA) and imaged using a confocal microscope.

### Confocal microscopy

Confocal images were gathered using a Carl Zeiss LSM 710 or Leica SP5 laser-scanning confocal microscope with a 63× objective. Z stacks were collapsed into projected images and merged using ImageJ (NIH, http://rsb.info.nih.gov/ij/). The color balance of the NMJ images was adjusted for clarity.

### Nerve conduction studies

Sciatic nerve conduction velocity was calculated by measuring the latency of compound motor action potentials recorded in the muscle of the left rear paw. The mice were anesthetized with 1% isofluorane and placed on a thermostatically regulated heating pad to maintain normal body temperature. Action potentials were produced by subcutaneous stimulation at the sciatic notch and at the ankle. For recording, the active needle electrode was inserted in the center of the paw and a reference electrode was placed in the skin between the first and second digits.

### Statistical analysis

Statistical tests were performed using GraphPad's Prism 5 software. Statistical significance was determined using a one-way ANOVA and a post hoc Tukey test for individual differences when appropriate. A threshold of p<0.05 was considered significant. The use of other tests is noted in the text. All results are presented as means ± SD.

## Supporting Information

Figure S1 GARS expression was assayed by western blot for all of the crosses performed in this study. Spinal cord and sciatic nerve tissues from two mice in each genotype were analyzed for GARS expression levels by western blot: β actin was used as a loading control.
(TIF)

Figure S2 Myelin thickness and axon diameter in motor nerves are not affected by transgene A or D. The diameter and myelin thickness of 100 axons were measured and averaged in each animal. The axon diameter data is the mean of the data presented as a cumulative histogram in Figure 2. Axon diameter averages were as follows: wild type = 3.7±0.3 (n = 6), WT; *TgA* = 3.6±0.3 (n = 6), WT; *TgD* = 3.7±0.4 (n = 6), *Nmf249/+* = 1.9±0.8 (n = 5), *Nmf249/+*; *TgA* = 2.0±0.9 (n = 5), and *Nmf249/+*; *TgD* 2.1±0.1 (n = 3). Myelin thickness averages were as follows: wild type = 1.05±0.10 (n = 6), WT; *TgA* = 1.13±0.13 (n = 6), WT; *TgD* = 1.20±0.08 (n = 6), *Nmf249/+* = 0.85±0.11 (n = 5), *Nmf249/+*; *TgA* = 0.92±0.12 (n = 5), and *Nmf249/+*; *TgD* 0.87±0.02 (n = 3).
(PDF)

Figure S3 Rescued mice weigh less than wild type, but muscles are not unduly atrophied. Wild-type mice at 8 weeks of age weighed 23.8±4.0 (n = 10) and *XM256/+* mice weighed 22.9±5.0 (n = 8). Neither of these was significantly different from *C201R/+* mice, which weighed 21.1±2.3 g (n = 4). Wild-type mice were heavier than the rescued *C201R/XM256*; *TgA* mice, which weighed 16.0±2.4 (n = 6), and *C201R/XM256*; *TgD* mice, which weighed 14.2±2.0 (n = 4) (p<0.01). (B) Rescued mice did not have significantly different ratios of muscle weight to body weight, a measure of muscle-specific atrophy. This shows that muscles in *C201R/XM256*; *TgA* and *C201R/XM256*; *TgD* mice are not smaller than muscles in *C201R/+* or controls relative to their total body mass. Two plantaris muscles were weighed and compared to the body weight of male mice in this cross. The muscle (mg) to bodyweight (g) ratios were as follows: the wild-type ratio was 1.69±0.14 (n = 4), the *XM256/+* ratio was 1.62±0.11 (n = 6), the *C201R/+* ratio was 1.33+0.09 (n = 4), the *C201R/XM256*; *TgA* ratio was 1.56±0.14 (n = 4), and the *C201R/XM256*; *TgD* ratio was 1.51±0.07 (n = 3), indicating that muscles are not smaller than anticipated for body weight, and that no neurogenic atrophy is evident.
(PDF)

Figure S4 Sensory neuropathy is similar in severity to motor neuropathy in *C201R/C201R*; *TgD*, *C201R/Nmf249*, and *C201R/Nmf249*; *TgD* mice. Femoral sensory nerve sections from wild type, *C201R/+*, and *Nmf249/+* littermate controls (A–C) are noticeably larger than sections from *C201R/C201R*; *TgD*, *C201R/Nmf249*, and *C201R/Nmf249*; *TgD* mice (D–F). The scale bar for A–F is in C and is 50 µm. G) Quantitative analysis confirmed this qualitative difference. Sensory nerve counts were as follows: 811±92 for wild-type mice (n = 7), 846±83 for *C201R/+* mice (n = 6), 865±79 for *Nmf249/+* mice (n = 5), 314±112 for *C201R/C201R*; *TgD* mice (n = 8), 289±74 for *C201R/Nmf249* mice (n = 4), and 244±102 for *C201R/Nmf249*; *TgD* mice (n = 5). *C201R/C201R*; *TgD*, *C201R/Nmf249*, and *C201R/Nmf249*; *TgD* mice had significantly fewer sensory axons than wild-type controls (p<0.001 for all three genotypes). These observations parallel the motor axon count differences seen in *C201R/C201R*; *TgD*, *C201R/Nmf249*, and *C201R/Nmf249*; *TgD* mice. Note that the change in the normal axon number in *Nmf249/+* sensory nerves is likely to be due to the younger age (P17) at which there mice were examined.
(TIF)
