# Supplementary figures and images for: Charcot-Marie-Tooth–Linked Mutant GARS Is Toxic to Peripheral Neurons Independent of Wild-Type GARS Levels
Source: PLoS Genet. 2011 Dec 1;7(12):e1002399. doi: 10.1371/journal.pgen.1002399 (PMC3228828; doi:10.1371/journal.pgen.1002399)

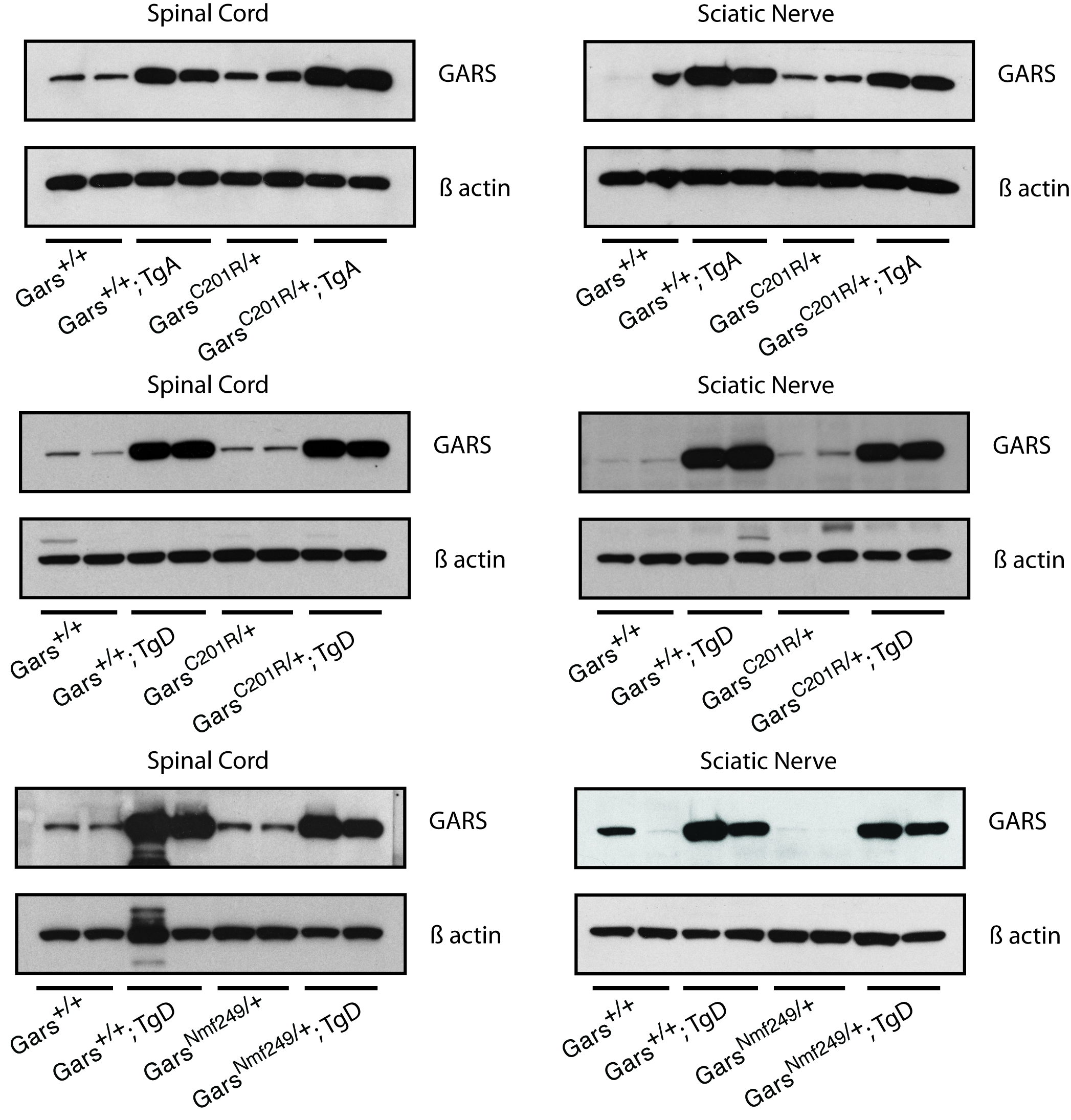

Supplement: Figure S1 — GARS expression was assayed by western blot for all of the crosses performed in this study. Spinal cord and sciatic nerve tissues from two mice in each genotype were analyzed for GARS expression levels by western blot: β actin was used as a loading control. (TIF) [file pgen.1002399.s001.tif]

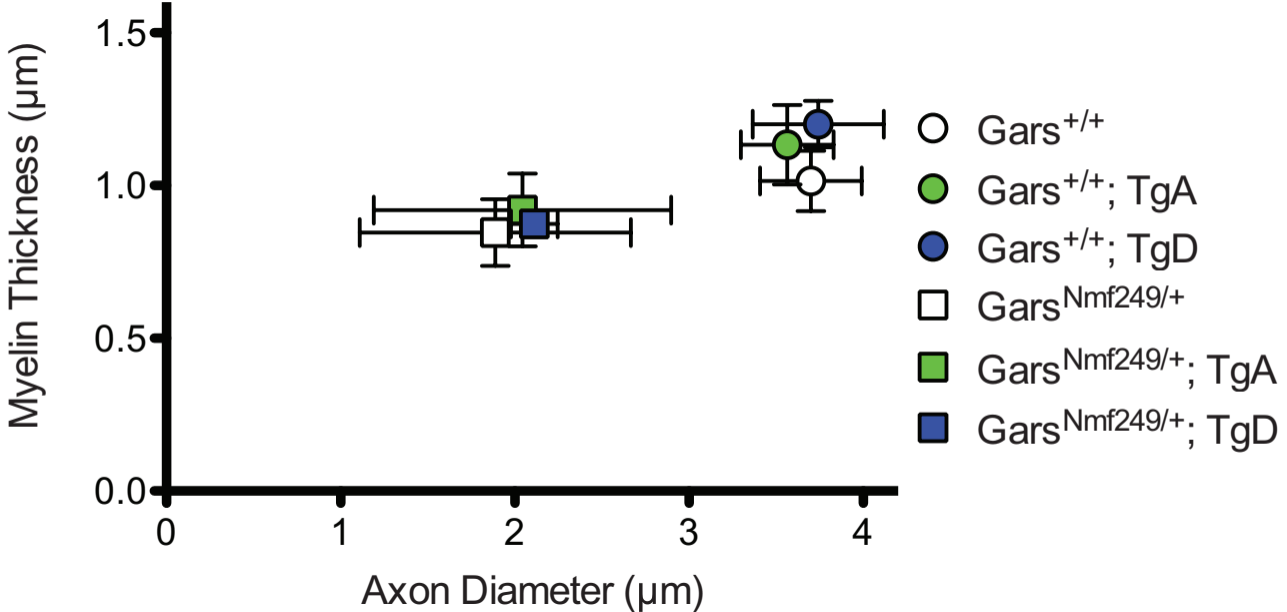

Supplement: Figure S2 — Myelin thickness and axon diameter in motor nerves are not affected by transgene A or D. The diameter and myelin thickness of 100 axons were measured and averaged in each animal. The axon diameter data is the mean of the data presented as a cumulative histogram in Figure 2. Axon diameter averages were as follows: wild type = 3.7±0.3 (n = 6), WT; TgA = 3.6±0.3 (n = 6), WT; TgD = 3.7±0.4 (n = 6), Nmf249/+ = 1.9±0.8 (n = 5), Nmf249/+; TgA = 2.0±0.9 (n = 5), and Nmf249/+; TgD 2.1±0.1 (n = 3). Myelin thickness averages were as follows: wild type = 1.05±0.10 (n = 6), WT; TgA = 1.13±0.13 (n = 6), WT; TgD = 1.20±0.08 (n = 6), Nmf249/+ = 0.85±0.11 (n = 5), Nmf249/+; TgA = 0.92±0.12 (n = 5), and Nmf249/+; TgD 0.87±0.02 (n = 3). (PDF) [file pgen.1002399.s002.pdf]

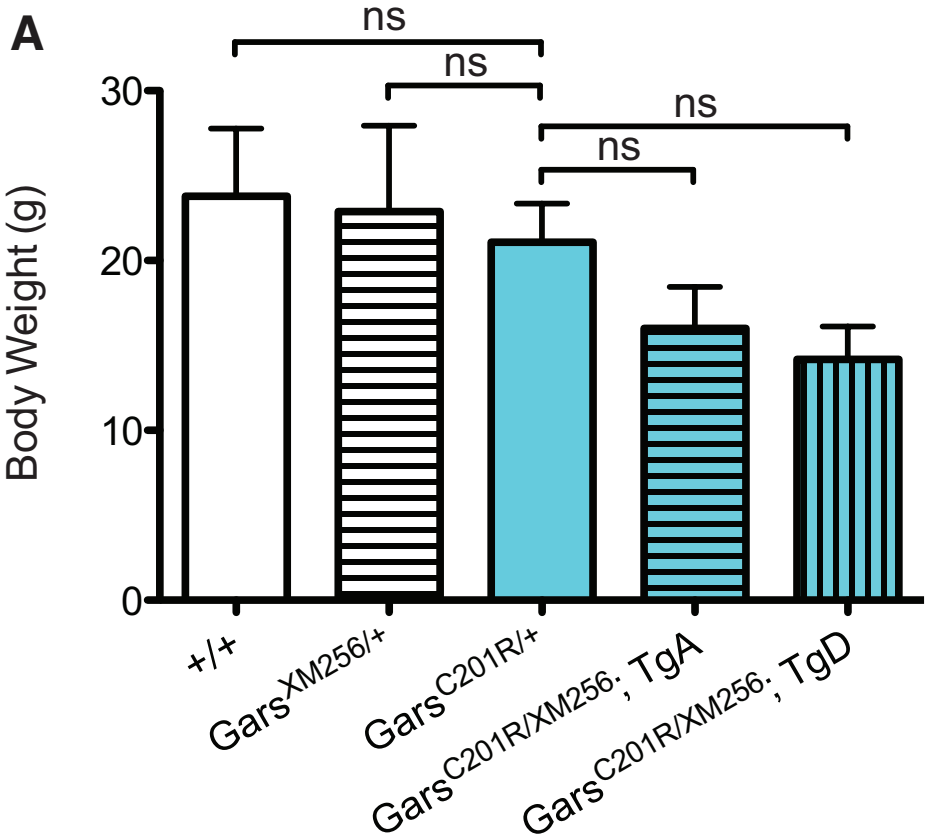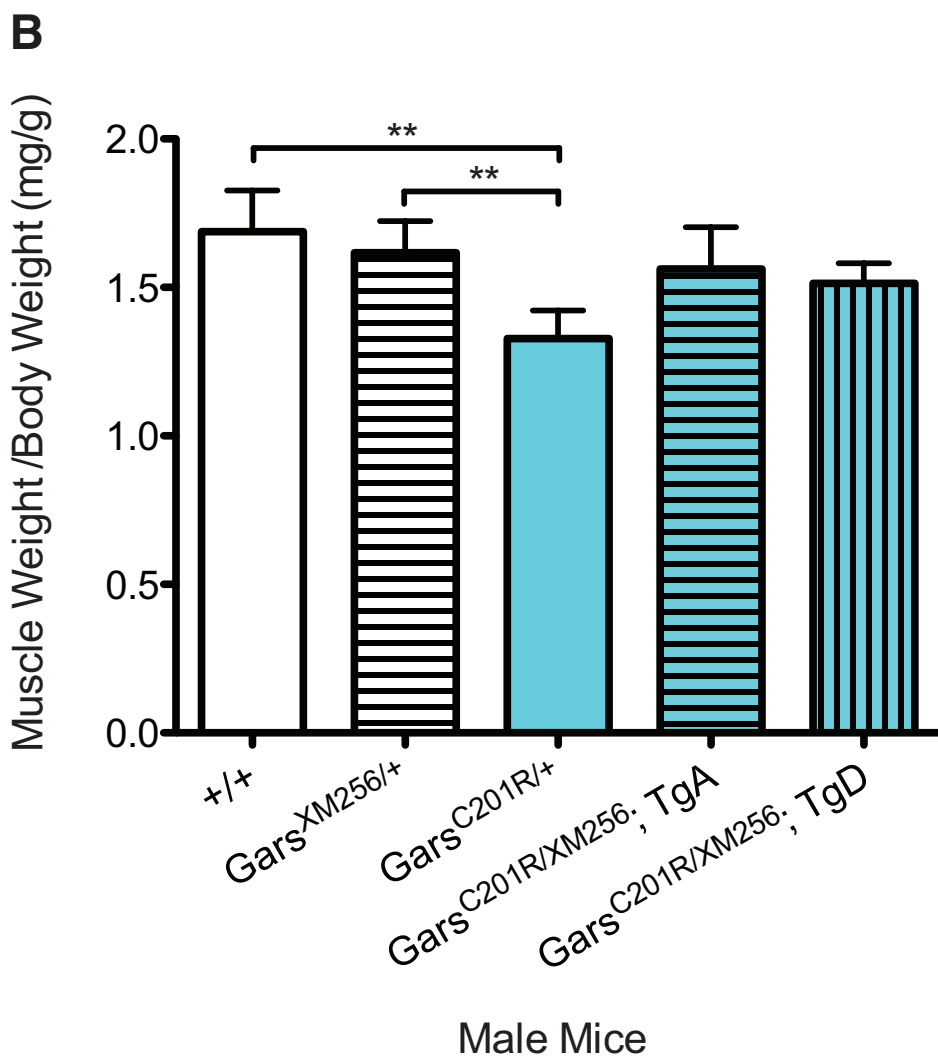

Supplement: Figure S3 — Rescued mice weigh less than wild type, but muscles are not unduly atrophied. Wild-type mice at 8 weeks of age weighed 23.8±4.0 (n = 10) and XM256/+ mice weighed 22.9±5.0 (n = 8). Neither of these was significantly different from C201R/+ mice, which weighed 21.1±2.3 g (n = 4). Wild-type mice were heavier than the rescued C201R/XM256; TgA mice, which weighed 16.0±2.4 (n = 6), and C201R/XM256; TgD mice, which weighed 14.2±2.0 (n = 4) (p<0.01). (B) Rescued mice did not have significantly different ratios of muscle weight to body weight, a measure of muscle-specific atrophy. This shows that muscles in C201R/XM256; TgA and C201R/XM256; TgD mice are not smaller than muscles in C201R/+ or controls relative to their total body mass. Two plantaris muscles were weighed and compared to the body weight of male mice in this cross. The muscle (mg) to bodyweight (g) ratios were as follows: the wild-type ratio was 1.69±0.14 (n = 4), the XM256/+ ratio was 1.62±0.11 (n = 6), the C201R/+ ratio was 1.33+0.09 (n = 4), the C201R/XM256; TgA ratio was 1.56±0.14 (n = 4), and the C201R/XM256; TgD ratio was 1.51±0.07 (n = 3), indicating that muscles are not smaller than anticipated for body weight, and that no neurogenic atrophy is evident. (PDF) [file pgen.1002399.s003.pdf]

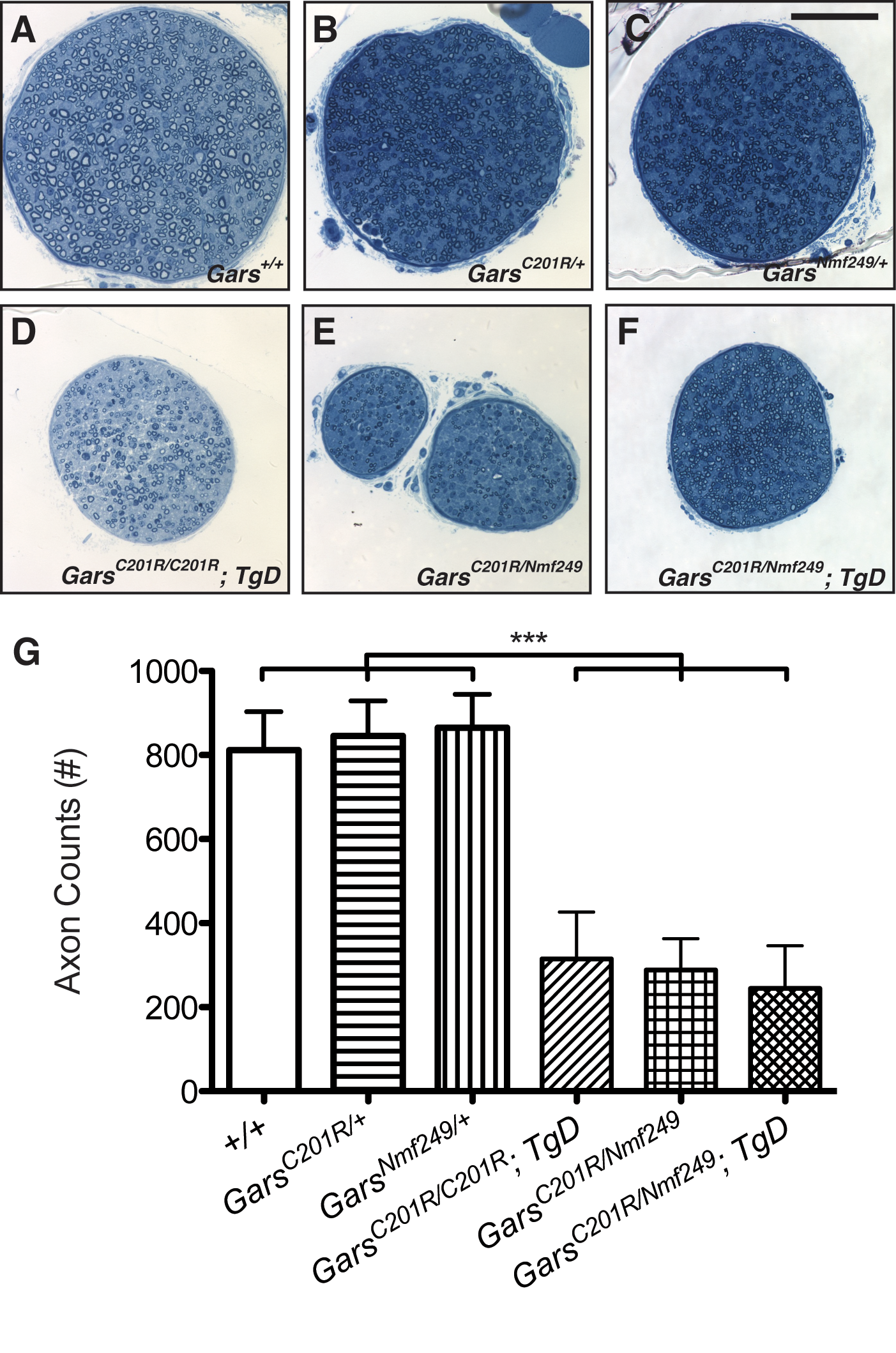

Supplement: Figure S4 — Sensory neuropathy is similar in severity to motor neuropathy in C201R/C201R; TgD, C201R/Nmf249, and C201R/Nmf249; TgD mice. Femoral sensory nerve sections from wild type, C201R/+, and Nmf249/+ littermate controls (A–C) are noticeably larger than sections from C201R/C201R; TgD, C201R/Nmf249, and C201R/Nmf249; TgD mice (D–F). The scale bar for A–F is in C and is 50 µm. G) Quantitative analysis confirmed this qualitative difference. Sensory nerve counts were as follows: 811±92 for wild-type mice (n = 7), 846±83 for C201R/+ mice (n = 6), 865±79 for Nmf249/+ mice (n = 5), 314±112 for C201R/C201R; TgD mice (n = 8), 289±74 for C201R/Nmf249 mice (n = 4), and 244±102 for C201R/Nmf249; TgD mice (n = 5). C201R/C201R; TgD, C201R/Nmf249, and C201R/Nmf249; TgD mice had significantly fewer sensory axons than wild-type controls (p<0.001 for all three genotypes). These observations parallel the motor axon count differences seen in C201R/C201R; TgD, C201R/Nmf249, and C201R/Nmf249; TgD mice. Note that the change in the normal axon number in Nmf249/+ sensory nerves is likely to be due to the younger age (P17) at which there mice were examined. (TIF) [file pgen.1002399.s004.tif]
